# Multidimensional evaluation of teaching strategies for pharmacology based on a comprehensive analysis involving 21,269 students

**DOI:** 10.3389/fphar.2023.1145456

**Published:** 2023-03-15

**Authors:** Chen-Lin Xiao, Huan Ren, Hui-Qing Chen, Wen-Hui Liu, Zhi-Ying Luo, Wen-Ru Li, Jian-Quan Luo

**Affiliations:** ^1^ Department of Pharmacy, The Second Xiangya Hospital, Central South University, Changsha, China; ^2^ Institute of Clinical Pharmacy, Central South University, Changsha, China; ^3^ Department of Pharmacy, Hunan Provincial People’s Hospital, The First Affiliated Hospital of Hunan Normal University, Changsha, China

**Keywords:** pharmacology education, network meta-analysis, teaching strategies, teaching methods, effectiveness

## Abstract

**Background:** Given the limitations of traditional pharmacology pedagogical method, diverse novel teaching methods have been widely explored. In this study, we performed a network meta-analysis (NMA) to evaluate the effects of different strategies in pharmacology education.

**Methods:** Literature databases were searched from their inception to November 2022, and the studies were screened according to predefined inclusion and exclusion criteria to extract important information. Outcomes, including theoretical test scores, experimental test scores, subjective test scores, satisfaction scores, and the proportion of satisfaction, were analyzed using R software (version 3.6.1) and STATA (version 15). The NMA was conducted with a random-effects model under the Bayesian framework to calculate odds ratios (ORs) or mean differences (MDs) with associated 95% credible intervals (95% CIs). Surface under the cumulative ranking curve (SUCRA) probability values were calculated to rank the teaching methods examined.

**Results:** A total of 150 studies involving 21,269 students were included. This NMA systematically evaluated 24 teaching strategies, such as problem-based learning (PBL), team-based learning (TBL), case-based learning (CBL) and flipped classrooms (FC), etc., The results of the NMA showed that, PBL combined with CBL was most likely to improve students’ theoretical and subjective test scores (SUCRA = 75.49 and 98.19%, respectively), TBL was most likely to improve the experimental test score (SUCRA = 92.38%) and the satisfaction score (SUCRA = 88.37%), while FC had the highest probability of being the best option for improving the proportion of satisfaction (SUCRA = 84.45%).

**Conclusion:** The current evidence indicates that TBL, PBL combined with CBL, and FC might be optimal strategies for pharmacology education since they have a more beneficial effect on students.

## 1 Introduction

The teaching of pharmacology is a challenging task as it involves the kinetics, dynamics and application of drugs (Haranath, 2016; White et al., 2017). The pharmacology education has long been dominated by the traditional method, lecture-based learning (LBL) (Faisal et al., 2016; Zeng et al., 2020). However, a typical LBL method does not perform well in helping students improve academic performance and cultivate comprehensive ability (Fu et al., 2022; Peng et al., 2022). Hence, with the continuous reform of pharmacology education, steps have been taken to ameliorate the situation (Curro et al., 2016; Engels, 2018; Alsanosi, 2022).

Many novel teaching methods (Sukhlecha et al., 2016; Palappallil et al., 2019) have been proposed to provide valuable pharmacology teaching for students in recent years. The most widely explored novel methods are problem-based learning (PBL) (Sengupta and Sur, 2021), team-based learning (TBL) (Nguyen et al., 2016; El-Banna et al., 2020), and case-based learning (CBL) (Kaur et al., 2020; Chiranjeevi et al., 2022). During the latest decade, with the development of computer software and mobile apps, flipped classrooms (FC) (Lockman et al., 2017) and micro classes (MC) (Wu et al., 2022) have been used widely in pharmacology education. The novel teaching methods were reported to be excellent in improving students’ academic performance, scientific literacy and subjective enthusiasm (El-Banna et al., 2017; Kennedy, 2019; Cheng et al., 2021). Furthermore, the novel methods promote students’ communication and mutual help. In this process, students share their learning experience, which is conducive to improving their academic performance and expression ability.

However, the effect of these novel methods was varied considerably across studies (Miller, 2003; Brinkman et al., 2021). Besides, the performance of these methods has not been compared in pharmacology education from different outcome measurements. Therefore, what kind of teaching methods should be selected troubles curriculum designers and educators. Since most studies compare novel methods with LBL, the direct comparisons of certain teaching methods cannot be done in traditional meta-analysis. Hence, in this study, we aimed to conduct a network meta-analysis by integrating available direct and indirect evidence to identify the optimal strategy for pharmacology teaching.

## 2 Materials and methods

This NMA was conducted following the Preferred Reporting Items for Systematic reviews and Meta-Analyses (PRISMA) extension statement to compare the effectiveness of different teaching methods in improving the pharmacology learning of students (Cornell, 2015; Hutton et al., 2015; Rethlefsen et al., 2021). This NMA did not require ethical approval, because the study only collected data from each study and did not disclose patient information.

### 2.1 Search strategy

Two reviewers (CX and HC) independently searched four electronic databases, including PubMed, EMBASE, China National Knowledge Infrastructure, and Chinese Wanfang Database, from database inception to 30 November 2022. A search strategy was developed, as illustrated in Supplementary Table S1 using PubMed as an example. The search terms mainly comprised three aspects: 1) “teaching method” OR “problem-based learning” OR “PBL” OR “team-based learning” OR “TBL” OR “task-driven learning” OR “case-based learning” OR “CBL” OR “active learning” OR “evidence-based medicine” OR “flipped classroom” OR “micro class” OR “blending learning” OR “mixed teaching” OR “BOPPPS” OR “scaffolding teaching method” AND 2) “pharmacology” AND 3) “comparative study” OR “comparison” OR “randomized control” OR “randomization”. Furthermore, corresponding modifications were made to accommodate the requirements of different databases. In addition, the reference lists of relevant reviews or meta-analyses were manually screened to identify potentially eligible publications.

### 2.2 Inclusion and exclusion criteria

All retrieved literature was managed by Zotero software. We included studies that met the following inclusion criteria: 1) Students receiving pharmacology education, regardless of age, gender, ethnicity, nationality, degree, and major; 2) Comparisons of novel teaching methods with another teaching method or LBL; novel teaching methods: PBL, TBL, CBL, active learning (AL), evidence-based medicine (EBM), FC, MC, task driven learning (TDL), computer-based learning (CoBL), blending learning, mixed teaching mode (MTM), BOPPPS (bridge-in, objective, pre-assessment, participatory learning, post-assessment, and summary), and scaffolding teaching method (STM); 3) Reports including the outcome measurements of pharmacology learning; 4) Randomized controlled trials (RCTs) and quasi-RCTs; and 5) Either English or Chinese publications. The exclusion criteria were as follows: 1) Overlapping publications; 2) Single-arm studies; 3) unavailability of full-text or valid data; 4) Subjects other than pharmacology; 5) Reviews, conference abstracts, case reports and meta-analysis.

### 2.3 Outcome measures and data extraction

The outcome was the effectiveness of students’ pharmacology learning, which was measured by the test scores and the incidence of students who endorsed the effectiveness of the teaching method in improving their comprehensive competency from the questionnaires. 1) Effect of different teaching method on improving test scores: the theoretical test score, the experimental test score, and the subjective test score. 2) Students’ satisfaction with different teaching methods: the satisfaction score, and the proportion of satisfaction.

We extracted the following important information from all eligible articles: name of the first author, publication year, sample size (intervention group and control group), the characteristics of the students, teaching method of intervention group and control group, and outcome measurements.

### 2.4 Quality assessment

We used the Cochrane Collaboration’s tool to assess the following biases of the included studies: Selection bias (random sequence generation and allocation concealment), performance bias, detection bias, attrition bias, reporting bias, and other bias (Higgins et al., 2011). Each item was classified as high risk, low risk, unclear risk, or not applicable. The graph was synthesized in R software (version 3.6.1) by loading the ggplot2 package.

### 2.5 Data synthesis and statistical analysis

We conducted NMA to estimate the rank of different teaching methods in pharmacology learning by combining direct and indirect comparisons. The analysis of 5 outcome measurements was statistically analyzed separately in our study. Outcomes were summarized as odd ratios (ORs) or mean differences (MDs) with associated 95% credible intervals (95% CIs), which were derived under the Bayesian framework using the random-effects model and calculated by Markov Chain Monte Carlo (MCMC) simulation (Warn et al., 2002). We used the random-effects model to obtain more conservative conclusions regardless of heterogeneity (Hu et al., 2020; Harrer et al., 2021). An MD > zero indicates a higher score of the intervention teaching strategy, and 95% CI that did not include zero was considered statistically significant. An OR > 1 indicates a higher incidence of students who endorsed the effectiveness of the intervention teaching method, and 95% CI that did not include 1.0 was considered statistically significant. In addition, the surface under the cumulative ranking curve (SUCRA) is used to estimate the probability of ranking each teaching strategy. The larger the area under the curve, the higher the ranking (Salanti et al., 2011).

The node-splitting method was used to detect the inconsistency between direct and indirect comparisons (Higgins et al., 2012; van Valkenhoef et al., 2016). As there is no indirect comparison and direct comparison at the same time, there is no need to identify inconsistencies for the subjective test score and the satisfaction score.

In order to compare the effects of different methods in the outcome indicators at the same time, we draw a figure displaying the SUCRA probability of the methods reported in at least 4 outcome indicators with GraphPad Prism 8 software (GraphPad, San Diego CA, United States). Potential publication bias was assessed by funnel plots and Egger’s test (Egger et al., 1997). A *p*-value >0.05 suggested no publication bias in the included studies. Besides, we employed the Grading of Recommendations Assessment, Development and Evaluation (GRADE) Working Group approach to assess the strength of evidence (Guyatt et al., 2011; Puhan et al., 2014). All analysis results were generated in R software (version 3.6.1) by loading the GeMTC package and calling the JAGS software (version 4.2.0) (van Valkenhoef et al., 2012). We generated network graphs in Stata (version 15) to elucidate treatments belonging to direct or indirect comparisons.

## 3 Results

### 3.1 Study selection

**FIGURE 1:**
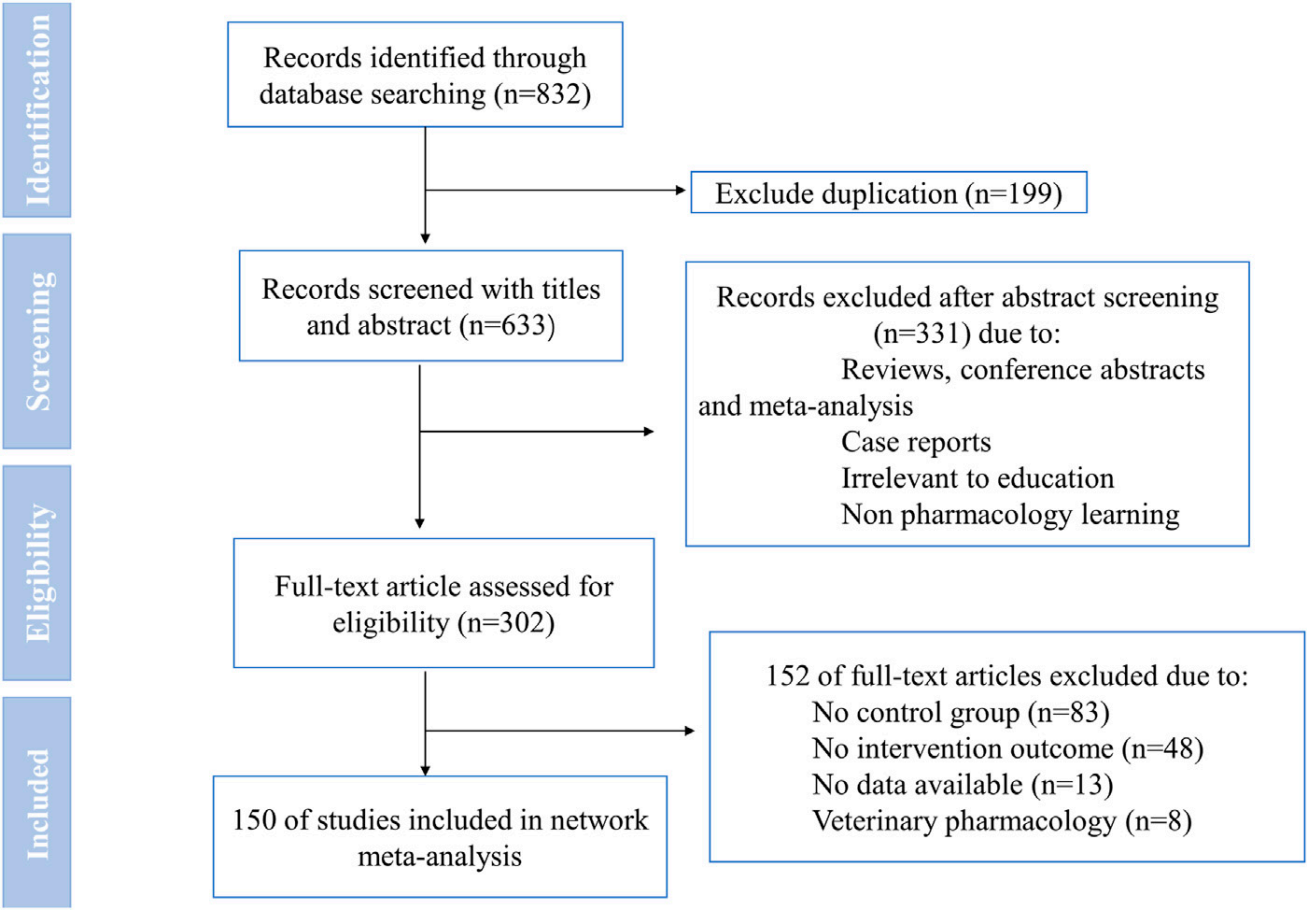
PRISMA flow diagram.

From the primary literature search, we identified 832 studies, of which 633 remained after the removal of duplicates. Next, a total of 331 articles were adopted after the title and abstract screening. Then, the full text of the remaining 302 articles was screened. We excluded 152 articles that either did not have a control group, did not have intervention outcomes, did not have available data, or belonged to veterinary pharmacology in the full-text eligibility stage. According to the pre-defined inclusion and exclusion criteria, 150 RCTs were finally included for NMA. Further details of the literature screening process are shown in Figure 1. The enrolled studies were published between 1989 and 2022, among which 120 (80.0%) were published in the last decade.

### 3.2 Characteristics of included studies

This NMA included 150 randomized controlled studies enrolling 21,269 students. The detailed characteristics of the studies are shown in Supplementary Table S2. In the included studies, the majors of students are mainly medicine (77/150, 51.3%), pharmacy (42/150, 28.0%) and nursing (31/150, 20.6%). As for the degree, 62.0% of students (13196/21269) are studying at the undergraduate stage (98/150 studies). Besides, the teaching method of the control group in 97.3% of studies (146/150) is LBL.

Regarding outcome variables, 134 studies reported theoretical, and 20 reported experimental test scores. Test scores for subjective questions were reported in 19 studies. In addition, questionnaires were employed in some studies to assess students’ preferences for teaching methods. The proportion of satisfaction and the satisfaction score were reported in 50 studies and 31 studies, respectively.

### 3.3 Quality of included studies

**FIGURE 2:**
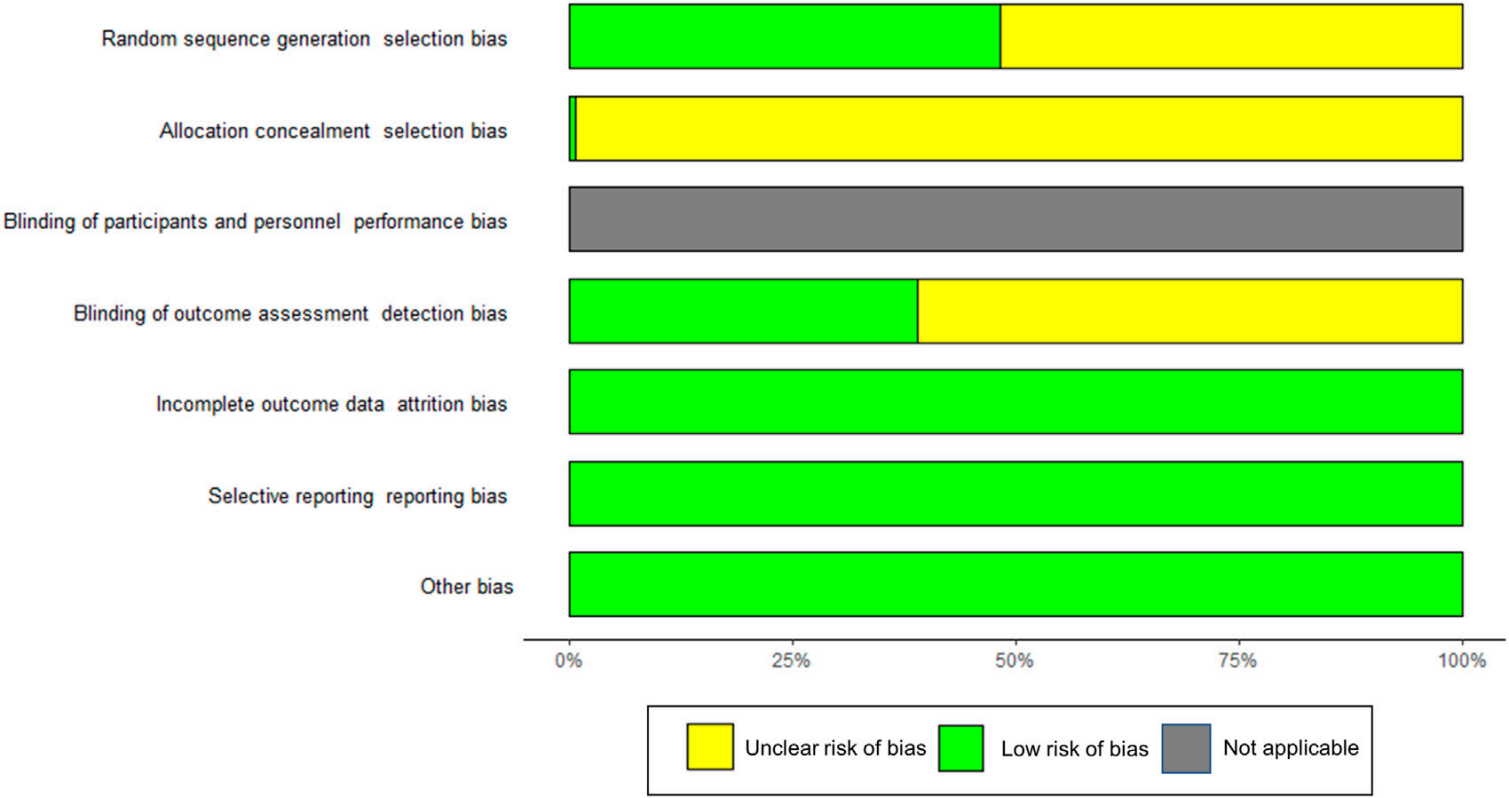
Risk assessment of bias using the Cochrane risk of bias. Risk of bias items of all included studies are indicated as the percentages. Green = low risk of bias, yellow = unclear risk of bias, gray = not applicable.

Using the Cochrane risk-of-bias (ROB) tool for quality assessment, Figure 2 presents a summary of assessed outcomes for the 150 included studies. A total of 83 studies employed an adequate method of random sequence generation and were rated as low risk. In addition, 2 studies reported the detailed allocation concealment procedure and were rated as low risk. Given the characteristics of teaching study, the students and teachers could not be blinded. Thus, the performance bias was not applicable. As for the detection biases, 59 studies implemented the blind method in outcome assessment and were rated as low risk. The remaining were judged as unclear risks. All the studies had complete data, and hence the attrition bias was assessed as low risk. The individual assessment of each study was presented in Supplementary Figure S1.

### 3.4 Effect of different teaching methods on improving test score

#### 3.4.1 The theoretical test score

**FIGURE 3:**
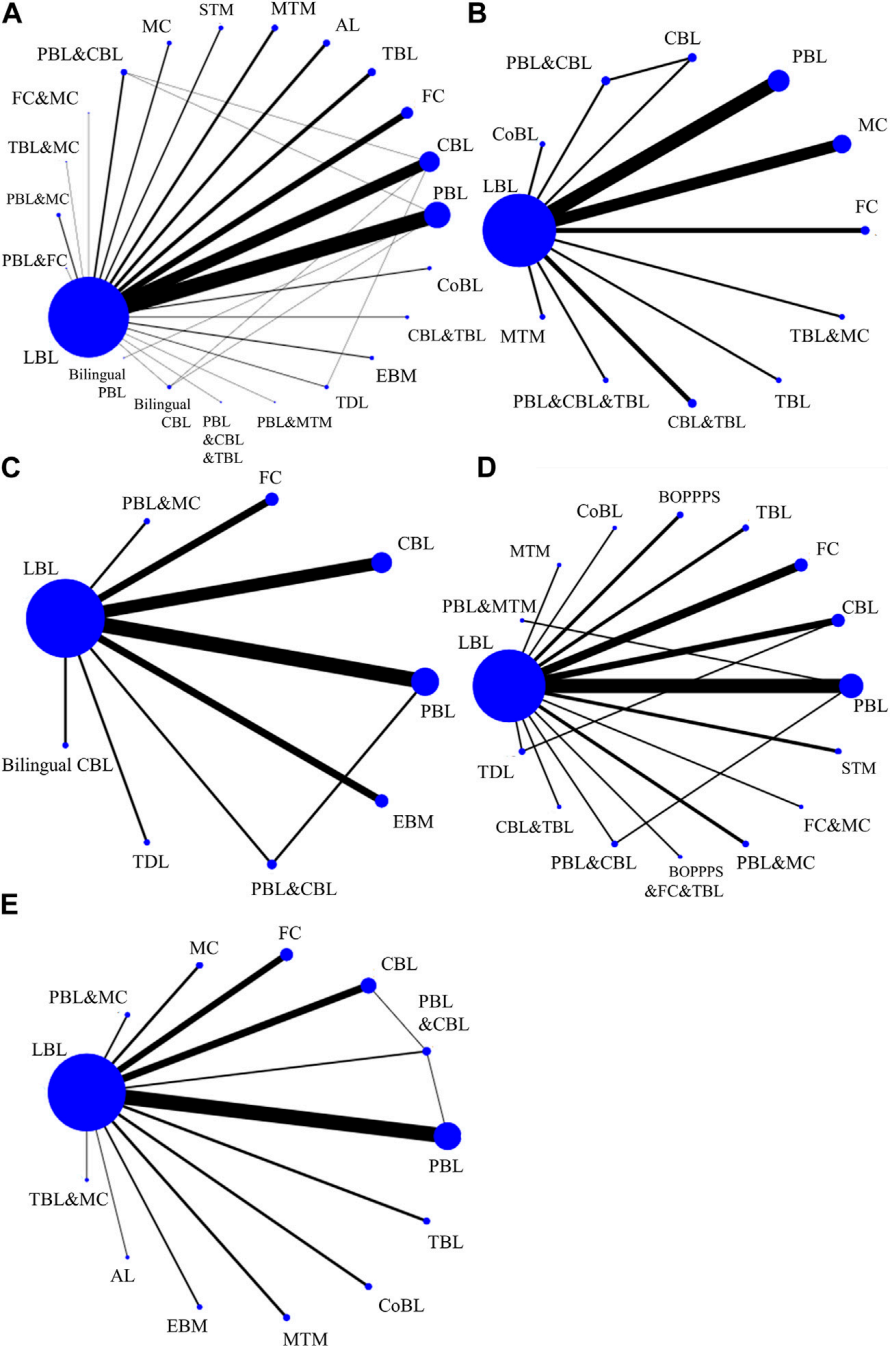
Network of teaching strategies in the Bayesian network meta-analysis. **(A)** The theoretical test score. **(B)** The experimental test score. **(C)** The subjective test score. **(D)** The satisfaction score. **(E)** The proportion of satisfaction. AL, active learning; BOPPPS, bridge-in, objective, pre-assessment, participatory learning, post-assessment, and summary; CBL, case-based learning; CoBL, computer-based learning; EBM, evidence-based medicine; FC, flipped classrooms; LBL, lecture based learning; MC, micro classrooms; MTM, mixed teaching mode; PBL, problem-based learning; STM, scaffolding teaching method; TBL, team-based learning; TDL, task driven learning.

The theoretical test score directly reflects the teaching effectiveness of each method on students and serves as the main outcome index. Among 150 studies, 134 studies involving 19,730 participants reported the theoretical test score. Methods of PBL (*n* = 42), CBL (*n* = 30), TBL (*n* = 10), FC (*n* = 17), MC (*n* = 4), AL (*n* = 8), EBM (*n* = 3), MTM (*n* = 7), TDL (*n* = 2), STM (*n* = 4), bilingual CBL (*n* = 1), CoBL (*n* = 3), CBL&TBL (CBL combined with TBL, *n* = 2), PBL&FC (PBL combined with FC, *n* = 1), FC&MC (FC combined with MC, *n* = 1), TBL&MC (TBL combined with MC, *n* = 1), PBL&MTM (PBL combined with MTM *n* = 1), PBL&CBL (PBL combined with CBL, *n* = 5), PBL&CBL&TBL (PBL combined with CBL and TBL, *n* = 1), and PBL&MC (PBL combined with MC, *n* = 3), were included. Details of other comparisons are shown in the network diagram (Figure 3A).

**FIGURE 4:**
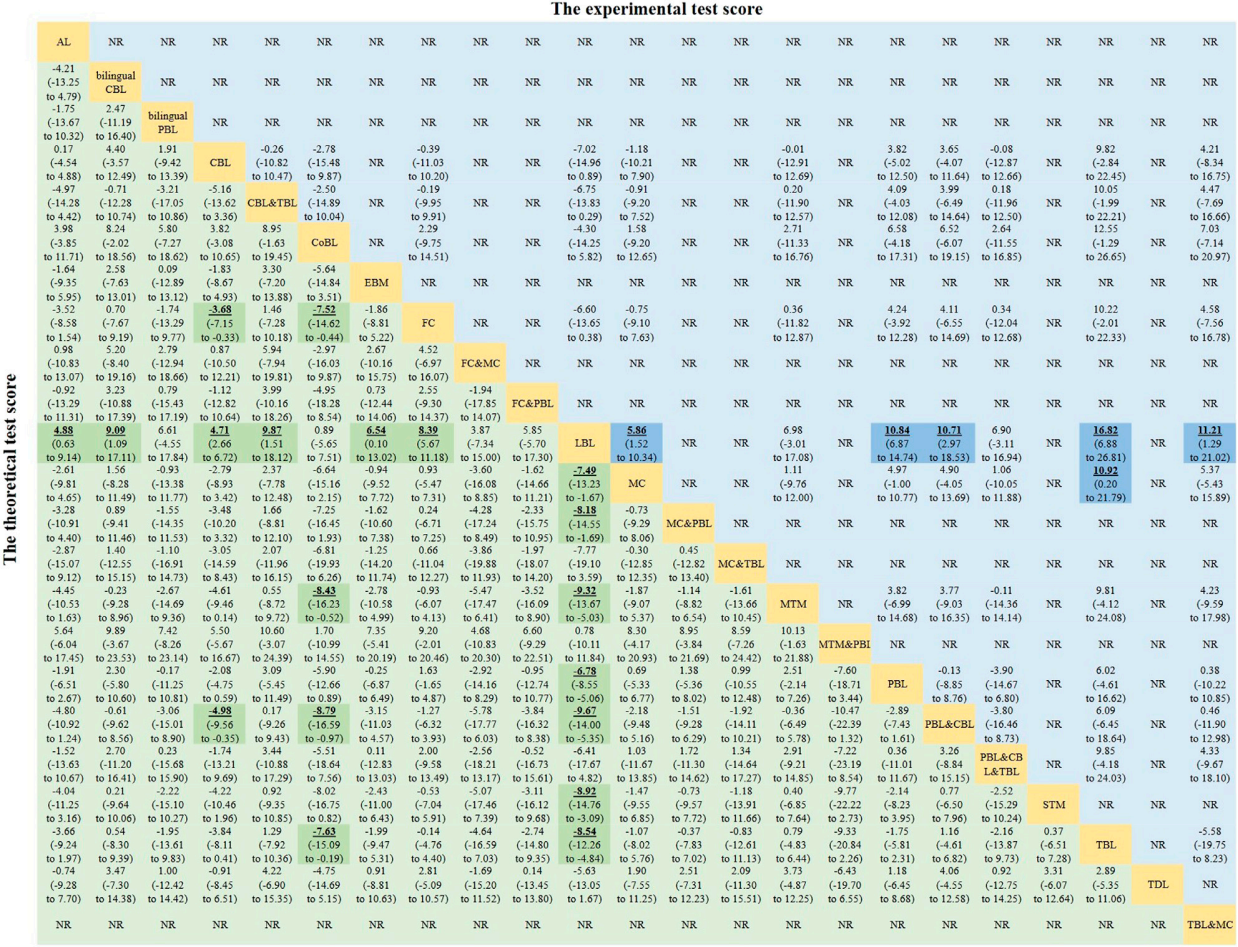
Mean differences of the theoretical test score (lower triangle) and the experimental test score (upper triangle). Data with mean differences represent the comparison of row-defining method *versus* column-defining method. Data in parentheses are the 95% credible intervals. Mean differences more than zero favour the column-defining method. Significant results are highlighted in bold, underline, and background fill. NR represents this indicator was not reported in this method among the included studies.

MD values generated by NMA are shown in Figure 4 (the lower triangle). Theoretical test scores were significantly higher in students learning with 13 novel methods, such as PBL&CBL (MD 9.67, 95% CI 5.35–14.00), TBL (MD 8.54, 95% CI 4.84–12.26), and FC (MD 8.39, 95% CI 5.67–11.18), etc., than in those receiving LBL teaching method. However, the impact of the other 8 novel methods on students’ theoretical scores was not significant. Besides, students receiving 4 methods, including PBL&CBL (MD 8.79, 95% CI 0.97–16.59), MTM (MD 8.43, 95% CI 0.52–16.23), TBL (MD 7.63, 95% CI 0.19–15.09), and FC (MD 7.52, 95% CI 0.44–14.62), had higher theoretical scores than those learning with CoBL method. Moreover, theoretical scores were significantly higher in students learning with FC and PBL&CBL than those receiving CBL method.

The results of the SUCRA rankings and probability values (Table 1), after ranking the effects of the teaching interventions, indicated that PBL&CBL was most likely to improve theoretical test scores in pharmacology learning (probability, 75.49%). Importantly, there are no inconsistencies in the direct and indirect comparisons between Bilingual CBL and CBL method (*p*-value = 0.623), LBL method (*p*-value = 0.633), CBL method and LBL method (*p*-value = 0.460), PBL&CBL method (*p*-value = 0.191), TDL method (*p*-value = 0.940), PBL&CBL method and LBL method (*p*-value = 0.157), PBL method (*p*-value = 0.968) (Table 2). For the remaining comparisons, node-splitting analysis could not be applied because there was no closed loop in the network.

**TABLE 1:** Surface under the cumulative ranking probabilities analysis (SUCRA) results for five outcome indexes.

Teaching method	Theoretical test score (%)	Experimental test score (%)	Subjective test score (%)	Satisfaction score (%)	Proportion of satisfactory (%)
LBL	8.76	4.32	10.06	31.99	8.45
PBL	50.41	72.96	67.54	56.70	57.11
CBL	32.25	45.00	56.39	39.89	61.53
TBL	66.83	**92.38**	NR	**88.37**	83.21
FC	66.25	42.74	46.14	45.21	**84.45**
MTM	72.53	45.87	NR	46.65	40.93
MC	57.27	36.49	NR	NR	53.28
AL	35.62	NR	NR	NR	2.72
TDL	43.99	NR	53.54	36.69	NR
Bilingual CBL	68.16	NR	29.52	NR	NR
Bilingual PBL	50.74	NR	NR	NR	NR
STM	68.21	NR	NR	73.34	NR
PBL&CBL&TBL	49.83	45.45	NR	NR	NR
PBL&CBL	**75.49**	70.24	**98.19**	47.09	63.30
CBL&TBL	72.29	43.78	NR	41.04	NR
PBL&MTM	14.77	NR	NR	52.74	NR
CoBL	15.54	30.03	NR	38.69	25.66
EBM	50.13	NR	55.13	NR	39.89
PBL&MC	61.98	NR	33.50	40.57	56.61
TBL&MC	56.78	70.74	NR	NR	72.86
PBL&FC	46.34	NR	NR	NR	NR
FC&MC	35.85	NR	NR	40.68	NR
BOPPPS	NR	NR	NR	38.75	NR
BOPPPS&FC&TBL	NR	NR	NR	81.60	NR

The strategies ranking first are marked in bold. NR, represents this indicator was not reported in this method among the included studies.

AL, active learning; BOPPPS, bridge-in, objective, pre-assessment, participatory learning, post-assessment, and summary; CBL, case-based learning; CoBL, computer-based learning; EBM, evidence-based medicine; FC, flipped classrooms; LBL, lecture based learning; MC, micro classrooms; MTM, mixed teaching mode; PBL, problem-based learning; STM, scaffolding teaching method; TBL, team-based learning; TDL, task driven learning.

**TABLE 2:** Results of node-splitting analysis.

Comparison	Direct effect	Indirect effect	Network effect	*p*-value
**Theoretical test score**				
Bilingual CBL vs. CBL	−2.4 (−14.0–9.2)	−6.4 (−17.0–5.0)	−4.4 (−12.5–3.6)	0.623
Bilingual CBL vs. LBL	−11.0 (−22.0–0.1)	−7.1 (−19.0–4.7)	−9.1 (−17.1–−1.1)	0.633
CBL vs. LBL	−4.8 (−6.9–−2.7)	−1.1 (−11.0–8.7)	−4.7 (−6.7–−2.7)	0.460
CBL vs. PBL + CBL	12.0 (0.4–23.0)	3.6 (−1.6–8.7)	5.0 (0.4–9.6)	0.191
CBL vs. TDL	3.3 (−7.8–14.0)	2.7 (−8.7–14.0)	0.9 (−6.5–8.4)	0.940
LBL vs. PBL + CBL	7.7 (2.7–13.0)	17.0 (5.1–29.0)	9.7 (5.3–14.0)	0.157
PBL vs. PBL + CBL	3.7 (−4.1–12.0)	3.5 (−2.4–9.5)	2.9 (−1.6–7.4)	0.968
**Experimental test score**				
CBL vs. LBL	−9.1 (−19.0–1.1)	−3.0 (-17.0–11.0)	−7.0 (−15.0–0.9)	0.449
CBL vs. PBL + CBL	5.8 (−4.4–16.0)	−0.3 (−14.0–14.0)	3.7 (−4.1–11.6)	0.445
LBL vs. PBL + CBL	8.8 (−1.0–19.0)	15.0 (0.8–29.0)	10.7 (3.0–18.5)	0.451
**Proportion of satisfactory**				
CBL vs. LBL	0.07 (0.03–0.18)	0.51 (0.02–13.5)	0.08 (0.03–0.2)	0.240
CBL vs. PBL + CBL	3.7 (0.3–60.3)	0.6 (0.08–4.1)	1.1 (0.2–5.3)	0.247
LBL vs. PBL + CBL	6.0 (0.9–40.4)	54.6 (3.0–1096.6)	12.8 (3.0–59.2)	0.219
PBL vs. PBL + CBL	1.0 (0.07–13.5)	2.0 (0.2–20.1)	1.2 (0.3–5.9)	0.678

The effects are presented with MDs in the theoretical test score and the experimental score, and ORs in the proportion of satisfactory. Data in parentheses are the 95% credible intervals. When the *p*-value >0.05, it is considered that there is no significant difference in statistics between the effect of direct comparison and the effect of indirect comparison. As there is no indirect comparison and direct comparison at the same time, there is no need to identify inconsistencies for the subjective test score and the satisfaction score.

CBL, case-based learning; LBL, lecture based learning; MDs, mean differences; ORs, odds ratios; PBL, problem-based learning; TDL, task driven learning.

#### 3.4.2 The experimental test score

The experimental test score directly reflects the effectiveness of experimental pharmacology teaching. A total of 20 studies involving 2,986 participants reported this index. This NMA systematically evaluate methods of PBL (*n* = 6), CBL (*n* = 1), TBL (*n* = 1), FC (*n* = 2), MC (*n* = 5), MTM (*n* = 1), CoBL (*n* = 1), CBL&TBL (*n* = 2), PBL&CBL (*n* = 1). Details of the comparisons are shown in the network diagram (Figure 3B).

MD values generated by NMA are displayed in the upper triangle of Figure 4. Experimental test scores were significantly higher in students learning with TBL method than in those receiving MC (MD 10.92, 95% CI 0.20–21.79) or LBL (MD 16.82, 95% CI 6.88–26.81) method. In addition, experimental test scores were significantly higher in students learning MC (MD 5.86, 95% CI 1.52–10.34), PBL (MD 10.84, 95% CI 6.87–14.74), PBL&CBL (MD 10.71, 95% CI 2.97–18.53), or TBL&MC (MD 11.21, 95% CI 1.29–21.02) than in those receiving LBL teaching method. There was no significant difference in experimental test scores among students studying pharmacology with other methods.

After ranking the effects of each intervention, the results of the SUCRA ranking and probability values (Table 1) indicated that TBL (probability, 92.38%) was most likely to improve experimental test scores in pharmacology learning. Moreover, there are no inconsistencies in the direct and indirect comparisons between CBL and LBL method (*p*-value = 0.449), CBL method and PBL&CBL method (*p*-value = 0.445), LBL method and PBL&CBL method (*p*-value = 0.451) (Table 2).

#### 3.4.3 The subjective test score

A total of 19 studies involving 2,731 participants reported the test score of subjective questions. Methods of PBL (*n* = 6), CBL (*n* = 5), bilingual CBL (*n* = 1), FC (*n* = 3), EBM (*n* = 3), TDL (*n* = 1), MC&PBL (*n* = 1), and PBL&CBL (*n* = 1) were included. Details of the comparisons are shown in the network diagram (Figure 3C).

**FIGURE 5:**
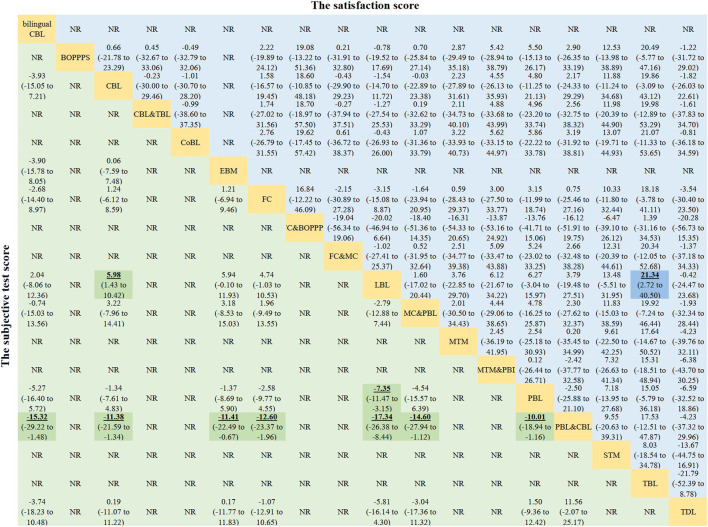
Mean differences of the subjective test score (lower triangle) and the satisfaction score (upper triangle). Data with mean differences represent the comparison of row-defining method *versus* column-defining method. Data in parentheses are the 95% credible intervals. Mean differences more than zero favour the column-defining method. Significant results are highlighted in bold, underline, and background fill. NR represents this indicator was not reported in this method among the included studies.

MD values generated by NMA are shown in the lower triangle of Figure 5. Subjective test scores were significantly higher in students learning PBL&CBL than in those receiving bilingual CBL (MD 15.32, 95% CI 1.48–29.22), CBL (MD 11.38, 95% CI 1.34–21.59), EBM (MD 11.41, 95% CI 0.67–22.49), FC (MD 12.60, 95% CI 1.96–23.37), PBL&MC (MD 14.60, 95% CI 1.12–27.94), PBL (MD 10.01, 95% CI 1.16–18.94) and LBL (MD 17.34, 95% CI 8.44–26.38) teaching methods. In addition, subjective test scores were significantly higher in students receiving PBL (MD 7.35, 95% CI 3.15–11.47) or CBL (MD 5.98, 95% CI 1.43–10.42) method than those learning pharmacology with LBL method. Besides, no significant difference was found in other comparisons. The results of the SUCRA (Table 1) indicated that PBL&CBL was most likely to improve subjective test scores in pharmacology learning (probability, 98.19%).

### 3.5 Students’ satisfaction with different teaching methods

#### 3.5.1 The satisfaction score

The satisfaction score directly reflects students’ subjective evaluation of different teaching methods. A total of 31 studies involving 3,165 participants reported methods of PBL (*n* = 8), CBL (*n* = 4), TBL (*n* = 2), FC (*n* = 5), BOPPPS (*n* = 2), MTM (*n* = 1), TDL (*n* = 1), STM (*n* = 2), CoBL (*n* = 1), CBL&TBL (*n* = 1), FC&BOPPPS (*n* = 1), FC&MC (*n* = 1), MC&PBL (*n* = 2), and PBL&CBL (*n* = 1). Details of the comparisons are shown in the network diagram (Figure 3D).

The satisfaction scores were significantly higher in students learning with TBL method than in those receiving LBL teaching method (MD 21.34, 95% CI 2.72–40.50). However, no significant difference was found in other comparisons. Other MD values generated by NMA are shown in the upper triangle of Figure 5. After ranking the effects of the teaching interventions (Table 1), TBL was most likely to improve satisfaction scores in pharmacology education (probability, 88.37%).

#### 3.5.2 The proportion of satisfaction

The proportion of satisfaction reflects students’ subjective evaluation of different teaching methods and served as another main outcome index. A total of 50 studies involving 6,559 students reported a proportion of satisfaction. Methods of PBL (*n* = 16), CBL (*n* = 8), TBL (*n* = 3), FC (*n* = 7), MC (*n* = 3), AL (*n* = 1), EBM (*n* = 2), MTM (*n* = 3), CoBL (*n* = 3), MC&TBL (*n* = 1), PBL&CBL (*n* = 2), and MC&PBL (*n* = 2), each was compared with LBL, were systematically evaluated. Details of the comparisons are shown in the network diagram (Figure 3E).

**FIGURE 6:**
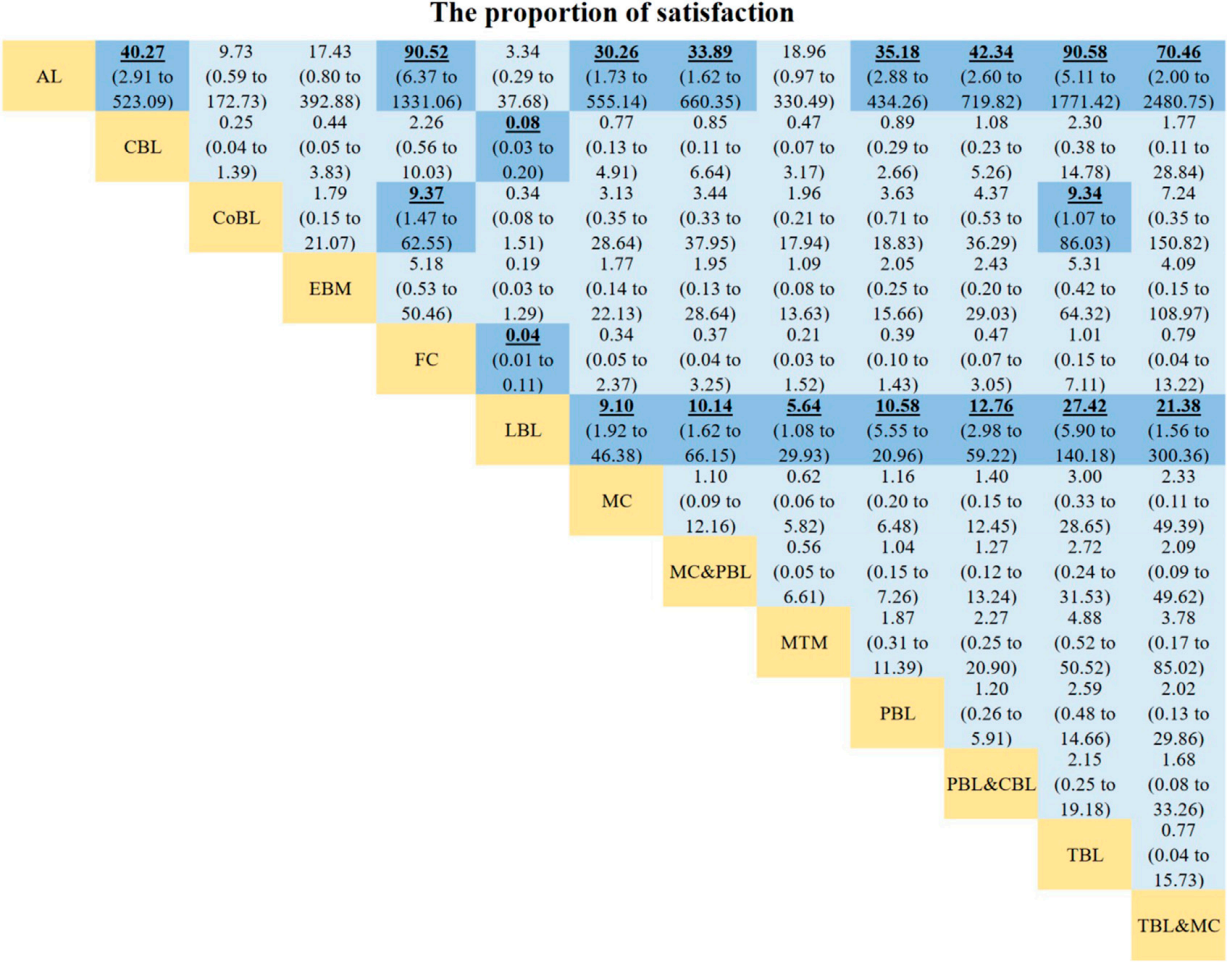
Odds ratios of the proportion of satisfaction. Data with odds ratios represent the comparison of row-defining method *versus* column-defining method. Data in parentheses are the 95% credible intervals. Odds ratios more than one favour the column-defining method. Significant results are highlighted in bold, underline, and background fill. NR represents this indicator was not reported in this method among the included studies.

OR values generated by NMA are displayed in Figure 6. The proportion of satisfaction was significantly higher in students learning CBL, FC (OR 27.11, 95% CI 9.15–88.23), MC, PBL&MC, PBL&CBL, TBL&MC, TBL (OR 27.42, 95% CI 5.90–140.18), PBL, or MTM than in those receiving LBL teaching method. Interestingly, compared with active learning, students are more satisfied with other learning methods (CBL, FC, MC, PBL&MC, PBL&CBL, TBL&MC, TBL, and PBL). In addition, students studying pharmacology with FC or TBL had a higher proportion of satisfaction than those studying with CoBL method. No significant difference was observed in other comparisons.

The results of the SUCRA rankings and probability values (Table 1), after ranking the effects of the teaching interventions, indicated that students were most satisfied with FC and TBL methods in pharmacology teaching relative to other teaching methods (probability, 84.45% and 83.21%, respectively).

Importantly, there are no inconsistencies in the direct and indirect comparisons between CBL and LBL method (*p*-value = 0.240), CBL method and PBL&CBL method (*p*-value = 0.247), LBL method and PBL&CBL method (*p*-value = 0.219), PBL method and PBL&CBL method (*p*-value = 0.678) (Table 2).

### 3.6 Grade of evidence

**FIGURE 7:**
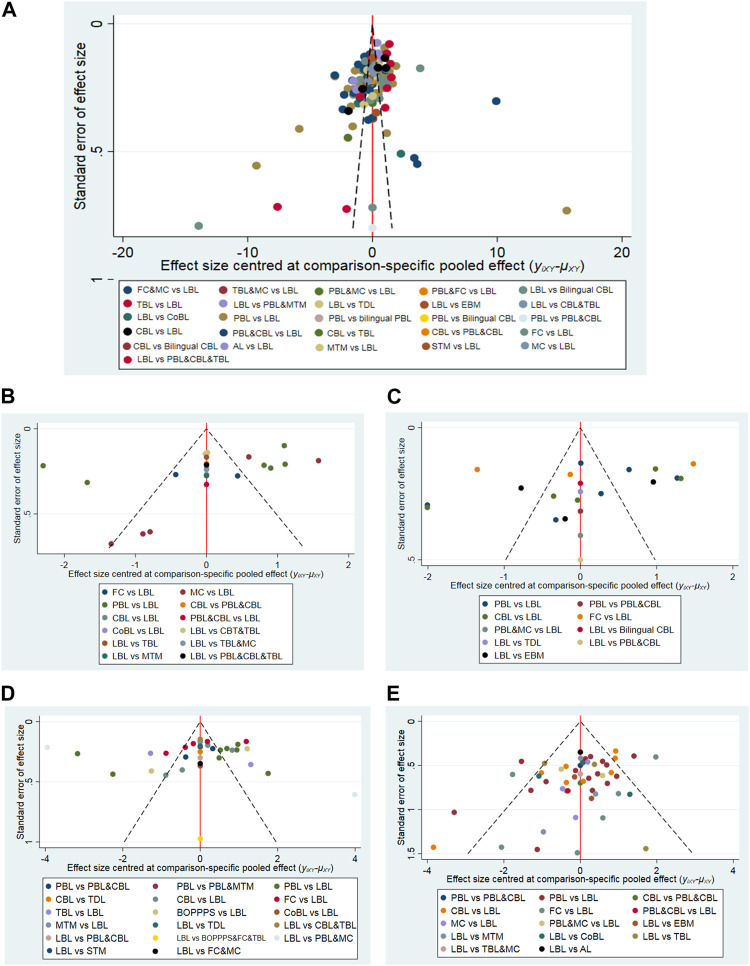
Funnel plots of the theoretical test score **(A)**, the experimental test score **(B)**, the subjective test score **(C)**, the satisfaction score **(D)**, and the proportion of satisfaction **(E)**.

The results of Egger regression test suggested no publication bias in the included studies. Besides, the funnel plots indicating no publication bias were found in the theoretical test score (Figure 7A, *p*-value = 0.655), the experimental test score (Figure 7B, *p*-value = 0.204), and the subjective test score (Figure 7C, *p*-value = 0.450), the satisfaction score (Figure 7D, *p*-value = 0.937), and the proportion of satisfaction (Figure 7E, *p*-value = 0.399).

There are no inconsistencies in the direct and indirect comparisons. Therefore, we have not downgraded the grade of evidence for publication bias and inconsistency. For study limitations, we downgraded by one level when the contributions from low RoB comparisons were less than 30% and contributions from moderate RoB comparisons were 70% or greater. In short, the certainty of the comparisons of the primary results (comparisons with statistical differences, which are highlighted in bold, underline, and background fill in Figures 4–6) was rated as “moderate”.

## 4 Discussion

**FIGURE 8:**
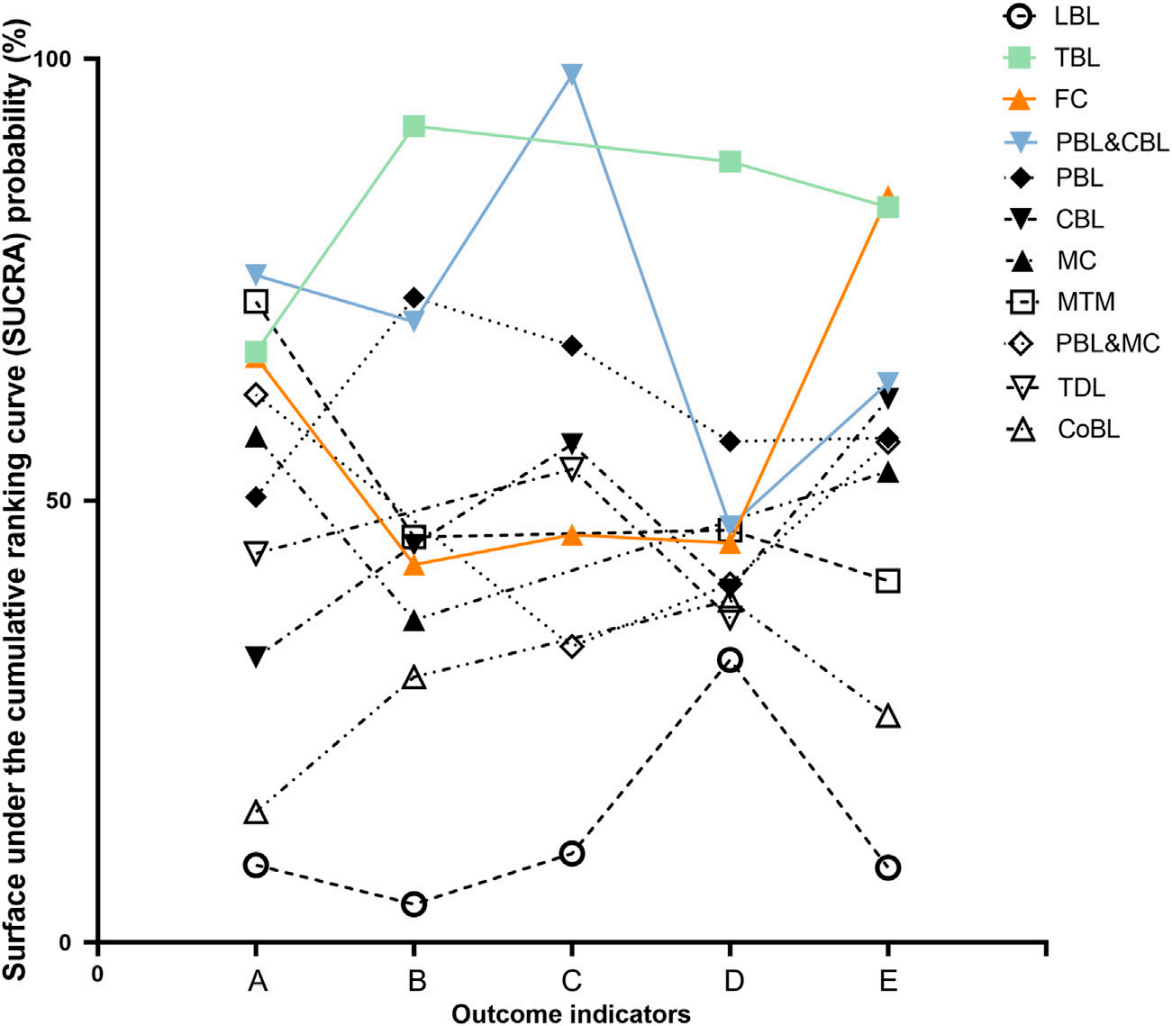
SUCRA probability of different teaching method in the outcome indicators. **(A)** The theoretical test score. **(B)** The experimental test score. **(C)** The subjective test score. **(D)** The satisfaction score. **(E)** The proportion of satisfaction.

To the best of our knowledge, this NMA is the first comprehensive data analysis assessing the effects of 24 pharmacology education strategies and collecting all available evidence from 150 RCTs involving 21,269 students. The pharmacology education is currently developing rapidly and is in a period of rapid development. Different pharmacology teaching methods and their combination are widely adopted worldwide and have achieved the desired teaching effect. However, the effectiveness of different teaching strategies has not been compared in pharmacology education. The NMA approach is more intuitive and has more information available than the classical meta-analysis. Thus, we carried out this NMA to supplement the optimal strategy and to strengthen additional insights for the development of pharmacology education in the future. Our findings point out that team-based learning (TBL), problem-based learning combined with case-based learning (PBL&CBL), and flipped classrooms (FC) are the most effective pharmacology teaching methods, although their ranking varies slightly among outcome measurements (Figure 8).

PBL&CBL is the optimal strategy when we use theoretical or subjective test scores to measure the effect of pharmacology education. Many studies (Michel et al., 2002; Liang et al., 2017) show that an integrated PBL programme may increase the competencies of medical students. This might be explained by PBL is intended to simulate active learning and enables students to work together and learn about a subject in the context of a real problem, such as case-based discussions. Besides, bringing an clinical element into pharmacology teaching could promote easy comprehension (Lau, 2004; Gupta et al., 2014). Hence, PBL&CBL enhances the students’ ability to analyze and learn the application of the pharmacology knowledge and can reflect on the educational experience gained through the cases/problems (Ghosh, 2007; Kamat et al., 2012; Mahajan et al., 2016). In addition, our results show that the theoretical scores of students receiving PBL&CBL method are higher than those learning with CBL alone. This suggests that when educators consider using CBL for teaching, they may get surprising pedagogical effect by designing some problems in advance and conducting targeted teaching.

Interestingly, the result of SUCRA probability (Figure 8) shows that the TBL method has performed well in the reported outcome indicators. When focusing on students’ satisfaction score and the experimental score, TBL is the optimal method. Meanwhile, TBL ranks second in the proportion of students’ satisfaction. These results suggest that TBL improves medical students’ interest in learning and their learning abilities. Students benefit from the implementation of TBL since TBL reduces the disparity in knowledge acquisition among them and encourages students to solve problems together (Burgess et al., 2014; Carrasco et al., 2021). As a result, students who may struggle with specific content have better performance with the help of group members and are more satisfied with TBL. Besides, Zgheib et al. reported that TBL was a cost-effective teaching technique, indicating that TBL was worth exploring (Zgheib et al., 2010).

FC is the best education strategy when it comes to the proportion of students’ satisfaction, and performs well in improving students’ theoretical test scores (Figure 8). Students who did not study hard before were forced to put in more effort with less readymade solutions provided by their teachers in FC. Besides, students master the core concepts of the course in the process of preparing the flipped classrooms, and thus get better academic feedback. Hence, FC method may improve medical students’ preference and their self-learning abilities (Rui et al., 2017; Zhang et al., 2019). However, among the other three outcome measurements, the SUCRA probabilities of FC were less than 50%, indicating that the performance of FC in improving students’ experimental and subjective test scores is worth further study.

The satisfaction of faculty and its associations with satisfaction of students are worth exploring. However, it was less frequently mentioned in studies on pharmacology education. Therefore, we were not able to carry out the corresponding NMA. Liu et al. (Liu et al., 2016) reported that the satisfaction score of the faculty in PBL (8.94 ± 0.42) and PBL&CBL (9.25 ± 9.36) were significantly higher than traditional lecture-based learning (LBL, 7.63 ± 0.33), which is similar to students’ preferences. Interestingly, the faculty rated active learning (AL) higher than LBL in 2 studies (4.5–4.8 vs. 4.1–4.4, and 3.9 vs. 4.1) (MacDougall, 2017; Kennedy, 2019). On the contrary, students’ satisfaction with AL was low. The reason might be that in the AL approach, the cost of time spent by the teacher is reduced, while the student needs to invest more time. In short, it is worth exploring how to find a balance in education.

To investigate the effect of academic background on the effectiveness of pharmacology teaching methods, we conducted a subgroup analysis on specialties (the medicine, pharmacy and nursing). The results (Supplementary Table S3) shows that academic background dose play a role to some extent in the effectiveness of different methods. For the theoretical test score, the first ranking method was influenced by the student’s academic background. When focusing on satisfaction, students in all the 3 majors were most satisfied with FC and TBL. The results imply that academic background had less of an impact on the satisfaction and more of an impact on the improvement of test score. However, this result may be influenced by factors such as the number of studies. On the one hand, the training objectives of the three majors are different. The credits taken up by pharmacology may be different. Thus, the interest and the effort put into pharmacology by students of these 3 majors may be different (Burks, 2022). On the other hand, pharmacology is a discipline that all the students in the three majors will need to master in their future careers.

Several limitations need to be taken into consideration. Due to the characteristics of the teaching process, the implementation of blinding in students and teachers was unrealizable. Thus, the performance bias in evaluating the quality of the literature was not applicable. The certainty of the comparisons of the primary results was rated as “moderate” because of risk of bias, which may carry a risk of overrating the effectiveness of the teaching strategies. In terms of this limitation, more rigorous RCTs with high certainty are needed to verify the results. However, the certainty of the evidence is currently acceptable for the exploration of pedagogical methods. Besides, the different baseline conditions of students, diverse test-design frameworks (Santiago et al., 2021; White et al., 2021) and the difference in teachers’ levels across all the included studies might contribute to the heterogeneity to some extent. In addition, most comparisons of treatments were based on indirect evidence, which might lead to a risk of imprecision. Hence, to increase the reliability of our results, we conducted an inconsistency analysis and risk of bias assessment to confirm the reasonability of this NMA. We did not do a formal cost-effectiveness analysis because of the lack of corresponding data. Pharmacology pedagogy studies focusing on the cost-effectiveness are worth conducting in the future to validate the feasibility of pedagogical methods from an economic perspective.

## 5 Conclusion

Overall, this NMA provides a comprehensive and integrated evaluation and summary of the effects of different pharmacology teaching methods. The current evidence indicates that TBL, PBL combined with CBL, and FC might have a more beneficial effect on students. It is imperative for pharmacology pedagogues to consider and promote these teaching strategies.

## Data Availability

The original contributions presented in the study are included in the article/Supplementary Material, further inquiries can be directed to the corresponding author.
