# Supplementary material for: Multidimensional evaluation of teaching strategies for pharmacology based on a comprehensive analysis involving 21,269 students
Source: Front Pharmacol. 2023 Mar 15;14:1145456. doi: 10.3389/fphar.2023.1145456 (PMC10050581; doi:10.3389/fphar.2023.1145456)
Supplement: Supplementary file 1 [file DataSheet1.pdf]

Supplementary Material

Multidimensional evaluation of teaching strategies for pharmacology based on a comprehensive analysis involving 21,269 students

Chen-Lin Xiao[1,2], Huan Ren[3], Hui-Qing Chen[1,2], Wen-Hui Liu[1,2], Zhi-Ying Luo[1,2], Wen-Ru Li[1,2], Jian-Quan Luo[1,2*]

[1]Department of Pharmacy, the Second Xiangya Hospital, Central South University, Changsha 410011, China

[2]Institute of Clinical Pharmacy, Central South University, Changsha 410011, China

[3]Department of Pharmacy, Hunan Provincial People's Hospital, The First Affiliated Hospital of Hunan Normal University, Changsha 410005, China

*** Correspondence:** Jianquan Luo: luojianquanxy@csu.edu.cn

1 Supplementary Tables

Table S1. Search strategy for the PubMed.

No.	Search terms
#1	education (mesh terms)
#2	teaching method (mesh terms)
#3	teaching strategy (mesh terms)
#4	(((((PBL[tiab]) OR (problem-based learning[tiab])) OR (case-based learning[tiab])) OR (CBL[tiab])) OR (TBL[tiab])) OR (team-based learning[tiab])
#5	((((((((((((((((flipped classroom[tiab]) OR (FC[tiab])) OR (MC[tiab])) OR (micro classroom[tiab])) OR (flipped class[tiab])) OR (flipped class[tiab])) OR (active learning[tiab])) OR (evidence-based medicine[tiab])) OR (EBM[tiab])) OR (TDL[tiab])) OR (task driven learning[tiab])) OR (computer-based learning[tiab])) OR (blending learning[tiab])) OR (mixed teaching mode[tiab])) OR (MTM[tiab])) OR (BOPPPS[tiab])) OR (scaffolding teaching method[tiab])) OR (STM[tiab])
#6	#1 or #2 or #3 or #4 or #5
#7	Groups [tiab]
#8	Trial [tiab]
#9	Comparison [tiab]
#10	Comparative study [tiab]
#11	Randomized [tiab]
#12	Randomly [tiab]
#13	Randomized controlled trial [pt]
#14	#7 or #8 or #9 or #10 or #11 or #12 or #13
#15	pharmacology [tiab]
#16	#6, #14 and #15

Table S2. Characteristics of the included studies.

Study	Student major	Student degree	Student equivalence	Sample size (interv./Cont.)	Interv	Cont	Outcome measurements
Sajjad et al 2021[1]	Nursing	Undergraduate	NR	118/140	FC	LBL	①
Lockman et al 2017[2]	Pharmacy	Doctor	NSSD	162/156	FC	LBL	①
Wu et al 2022[3]	Medicine	Undergraduate	NSSD	73/85	FC+MC	LBL	①④⑤
El-Banna et al 2017[4]	Nursing	Undergraduate	NSSD	36/40	FC	LBL	①④
Guo et al 2018[5]	Nursing	Junior college student	NSSD	42/42	FC	LBL	①⑤
Wang et al 2017[6]	Medicine	Graduate	NSSD	148/152	FC	LBL	①③⑤
Liang et al 2019[7]	Nursing	Undergraduate	NSSD	50/50	FC	LBL	①④
Wang ZC et al 2020[8]	Medicine	Undergraduate	NSSD	38/34	FC	LBL	①
Ge et al 2019[9]	Pharmacy	Undergraduate	NR	35/34	FC	LBL	②⑤
Wang JN et al 2020[10]	Pharmacy	Junior college student	NSSD	27/27	FC	LBL	①②⑤
Fu et al 2022[11]	Medicine	Undergraduate	NSSD	64/66	FC	LBL	①④
Zhao XM et al 2019[12]	Pharmacy	Undergraduate	NR	22/29	FC+PBL	LBL	①
Wong et al 2014[13]	Pharmacy	Undergraduate	NSSD	101/103	FC	LBL	①
Zhao et al 2018[14]	Nursing	Junior college student	NSSD	80/78	FC	LBL	①
Fan et al 2020[15]	Medicine	Junior college student	NSSD	106/108	FC	LBL	①③
Wang H et al 2021[16]	Medicine	Junior college student	NR	41/41	FC+MC	LBL	①④
Ding et al 2022[17]	Medicine	Undergraduate	NR	80/80	FC	LBL	①③
Jia et al 2020[18]	Nursing	Junior college student	NSSD	180/180	FC	LBL	①⑤
Bao et al 2018[19]	Medicine	Undergraduate	NSSD	38/38	FC	LBL	①④
Yang XY et al 2021[20]	Medicine	Undergraduate	NR	32/32	FC	LBL	①⑤
Qin Z et al 2020[21]	Pharmacy	Undergraduate	NSSD	10/10	FC+BOPPPS +TBL	LBL	④
Ma et al 2022[22]	Medicine	Undergraduate	NSSD	45/45	MC+PBL	LBL	①③④
Song et al 2018[23]	Medicine	Undergraduate	NSSD	56/56	MC+PBL	LBL	①④⑤
Qin X et al 2020[24]	Pharmacy	Undergraduate	NSSD	30/29	MC+PBL	LBL	①⑤
Bai et al 2018[25]	Medicine	Undergraduate	NR	60/59	MC	LBL	⑤
Yang et al 2016[26]	Nursing	Junior college student	NSSD	80/80	MC	LBL	①②⑤
Yang LJ et al 2021[27]	Pharmacy	Undergraduate	NSSD	58/56	MC	LBL	①②
Ruan et al 2019[28]	Nursing	Junior college student	NSSD	72/74	MC	LBL	②
Fan et al 2019[29]	Pharmacy	Undergraduate	NSSD	47/47	MC	LBL	①⑤
Qin et al 2018[30]	Nursing	Junior college student	NSSD	68/72	MC	LBL	①
Kaur et al 2020[31]	Medicine	Undergraduate	NSSD	47/47	CBL	LBL	①
Vora et al 2015[32]	Medicine	Undergraduate	NR	34/34	CBL	LBL	①
Li S et al 2014[33]	Medicine	Undergraduate	NR	96/89	CBL	LBL	①

Kamat et al 2012[34]	Medicine	Undergraduate	NSSD	82/61	CBL	LBL	①
Chiranjeevi et al 2022[35]	Medicine	Undergraduate	NSSD	30/30	CBL	LBL	①
Xia et al 2017[36]	Medicine	Undergraduate	NSSD	56/56	CBL	LBL	①③⑤
Chen MJ et al 2015[37]	Medicine	Undergraduate	NR	135/135	CBL	LBL	①⑤
Wang JH et al 2012[38]	Medicine	Undergraduate	NR	98/95	CBL	LBL	①
Yang et al 2020[39]	Pharmacy	Undergraduate	NSSD	77/83	CBL	LBL	①
Li et al 2022[40]	Pharmacy	Undergraduate	NSSD	29/29	CBL	LBL	①④
Chen et al 2014[41]	Nursing	Junior college student	NSSD	116/122	CBL	LBL	①⑤
Liu XJ et al 2015[42]	Medicine	Junior college student	NSSD	30/31	CBL	LBL	①④
Yuan et al 2013[43]	Pharmacy	Undergraduate	NR	40/40	CBL	LBL	①⑤
Sui et al 2009[44]	Nursing	Junior college student	NR	141/129	CBL	LBL	①
Huang W et al 2017[45]	Medicine	Junior college student	NR	35/35	CBL	LBL	①⑤
Zheng et al 2020[46]	Pharmacy	Undergraduate	NSSD	39/39	CBL	LBL	①②③
Song et al 2010[47]	Pharmacy	Undergraduate	NSSD	55/43	CBL	LBL	①④
Wang et al 2014[48]	Nursing	Undergraduate	NR	89/90	CBL	LBL	①③⑤
Yin et al 2013[49]	Pharmacy	Junior college student	NR	100/100	CBL	LBL	①
Zou et al 2014[50]	Pharmacy	Junior college student	NR	50/50	CBL	LBL	①⑤
Wang et al 2010[51]	Nursing	Undergraduate	NR	65/63	CBL	LBL	①③
Song et al 2014[52]	Pharmacy	Junior college student	NSSD	60/62	CBL	LBL	⑤
Liu P et al 2014[53]	Medicine	Undergraduate	NSSD	149/141	CBL	LBL	①
Wang GP et al 2017[54]	Nursing	Junior college student	NSSD	48/49	CBL	LBL	①③
Yang et al 2017[55]	Medicine	Undergraduate	NSSD	30/32	CBL	LBL	①
Nie et al 2006[56]	Medicine	Undergraduate	NR	60/60	CBL	LBL	①
Feldman et al 1989[57]	Medicine	Undergraduate	NR	25/15	CoBL	LBL	①
MacFadyen et al 1993[58]	Medicine	Undergraduate	NSSD	26/28	CoBL	LBL	①④⑤
Hahne et al 2005[59]	Medicine	Undergraduate	NSSD	70/97	CoBL	LBL	①
Joseph et al 2021[60]	Medicine	Undergraduate	NSSD	40/40	CoBL	LBL	②⑤
Cui et al 2013[61]	Medicine	Undergraduate	NR	25/25	bilingual PBL	PBL	①
Yu et al 2016[62]	Medicine	Undergraduate	NR	156/156	bilingual CBL	CBL	①
Huang et al 2015[63]	Medicine	Undergraduate	NSSD	51/53	bilingual CBL	LBL	①③
Liu et al 2016[64]	Pharmacy	Undergraduate	NSSD	165/77	PBL+CBL vs.PBL	LBL	①④
Croteau et al 2011[65]	Nursing	Undergraduate	NSSD	53/110	PBL+CBL	LBL	①
Song et al 2021[66]	Medicine	Junior college student	NSSD	52/52	PBL+CBL	CBL	①②⑤
Wang SC et al 2021[67]	Pharmacy	Junior college student	NSSD	60/60	PBL+CBL	LBL	①②⑤
Li C et al 2019[68]	Medicine	Undergraduate	NR	100/50	PBL+CBL/PBL	LBL	①③⑤
Li HY et al 2019[69] Li HY et al 2020[70]	Medicine	Junior college student	NSSD	201/203	CBL+TBL	LBL	①②④

Ma et al 2017[71]	Medicine	Undergraduate	NR	48/48	PBL+CBL+TBL	LBL	②
Dai et al 2016[72]	Pharmacy	Undergraduate	NR	184/92	PBL+CBL+TBL	LBL	①
James et al 2016[73]	Medicine	Undergraduate	NR	116/70	TBL	LBL	④⑤
Zhou et al 2020[74]	Medicine	Junior college student	NSSD	50/50	TBL	LBL	①
Xia et al 2022[75]	Medicine	Undergraduate	NSSD	96/94	TBL	LBL	②⑤
Wu et al 2017[76]	Medicine	Undergraduate	NSSD	20/20	TBL	LBL	①④
Li et al 2013[77]	Pharmacy	Junior college student	NSSD	60/60	TBL	LBL	⑤
Carrasco et al 2021[78]	Medicine	Undergraduate	NSSD	403/266	TBL	LBL	①
Palappallil et al 2019[79]	Medicine	Undergraduate	NR	96/49	TDL/CBL	LBL	①
McCartney et al 2020[80]	Pharmacy	Undergraduate	NSSD	104/69	TBL	LBL	①
El-Banna et al 2020[81]	Nursing	Undergraduate	NSSD	36/40	TBL	LBL	①
Zgheib et al 2010[82]	Medicine	Undergraduate	NR	13/12	TBL	LBL	①
Persky et al 2012[83]	Pharmacy	Doctor	/	154	TBL	LBL	①
Kim et al 2020[84]	Medicine	Undergraduate	NSSD	49/43	TBL	LBL	①
Nguyen et al 2016[85]	Medicine	Undergraduate	NR	33/36	TBL	LBL	①
Dong et al 2019[86]	Nursing	Undergraduate	NR	50/50	TBL+MC	LBL	①②⑤
Xiang S et al 2015[87]	Nursing	Junior college student	NSSD	114/58	PBL	LBL	①⑤
Liu LJ et al 2014[88]	Nursing	Junior college student	NR	108/108	PBL	LBL	①
Wang XL et al 2012[89]	Nursing	Junior college student	NSSD	124/63	PBL	LBL	①⑤
Yang et al 2008[90]	Nursing	Undergraduate	NR	26/30	PBL	LBL	①
Yang et al 2021[91]	Nursing	Undergraduate	NR	150/150	PBL	LBL	①②
Yang et al 2015[92]	Nursing	Junior college student	NSSD	50/50	PBL	LBL	①⑤
Li et al 2020[93]	Medicine	Undergraduate	NSSD	28/28	CoBL	LBL	⑤
Jia et al 2021[94]	Pharmacy	Junior college student	NSSD	48/48	PBL	LBL	①
Li J et al 2014[95]	Medicine	Junior college student	NSSD	186/302	PBL	LBL	①②
Liang T et al 2017[96]	Nursing	Undergraduate	NSSD	25/25	PBL	LBL	①③④
Zhao et al 2014[97]	Medicine	Junior college student	NSSD	151/133	PBL	LBL	①⑤
Xiang S(2) et al 2015[98]	Medicine	Junior college student	NSSD	92/92	PBL	LBL	①⑤
Wang et al 2008[99]	Medicine	Undergraduate	NR	189/120	PBL	LBL	①③
Cao et al 2011[100]	Medicine	Undergraduate	NSSD	61/65	PBL	LBL	①
Zhao et al 2015[101]	Pharmacy	Undergraduate	NR	70/70	PBL	LBL	①
Chen et al 2007[102]	Medicine	Undergraduate	NSSD	45/39	PBL	LBL	①
Liang et al 2008[103]	Nursing	Undergraduate	NSSD	89/90	PBL	LBL	①③⑤
Wang YL et al 2017[104]	Nursing	Junior college student	NSSD	60/58	PBL	LBL	①
Chen et al 2017[105]	Pharmacy	Junior college student	NR	51/55	PBL	LBL	①②
Miller et al 2003[106]	Nursing	Undergraduate	NR	10/12	PBL	LBL	①④

Sengupta et al 2021[107]	Medicine	Undergraduate	NSSD	46/45	PBL+MTM	PBL	①④
Liang T(2) et al 2017[108]	Medicine	Undergraduate	NSSD	40/40	PBL	LBL	①③④
Herzig et al 2003[109]	Medicine	Undergraduate	NR	55/57	PBL	LBL	①
Chen L et al 2015[110]	Medicine	Undergraduate	NR	82/63	PBL	LBL	①
Fu et al 2016[111]	Medicine	Undergraduate	NR	54/134	PBL	LBL	①⑤
Yang Y et al 2018[112]	Pharmacy	Junior college student	NR	32/32	PBL	LBL	①
Guo et al 2013[113]	Nursing	Junior college student	NR	27/29	PBL	LBL	①
Li W et al 2014[114]	Medicine	Junior college student	NSSD	40/40	PBL	LBL	①⑤
Zhou et al 2012[115]	Pharmacy	Undergraduate	NR	34/34	PBL	LBL	①
Huang W(2) et al 2017[116	Medicine	Junior college student	NR	40/40	PBL	LBL	①⑤
Li et al 2011[117]	Pharmacy	Undergraduate	NR	54/54	PBL	LBL	②
Song et al 2015[118]	Pharmacy	Undergraduate	NSSD	90/93	PBL	LBL	①⑤
Qin et al 2012[119]	Medicine	Undergraduate	NR	40/40	PBL	LBL	⑤
Jia et al 2013[120]	Pharmacy	Junior college student	NSSD	45/46	PBL	LBL	①②⑤
Gao et al 2015[121]	Pharmacy	Undergraduate	NSSD	60/58	PBL	LBL	①②⑤
Yang M et al 2018[122]	Medicine	Undergraduate	NSSD	30/30	PBL	LBL	①④
Song et al 2008[123]	Medicine	Undergraduate	NR	85/31	PBL	LBL	①⑤
Li et al 2017[124]	Pharmacy	Undergraduate	NSSD	98/96	PBL	LBL	①
Huo et al 2016[125]	Medicine	Undergraduate	NSSD	56/57	PBL	LBL	①⑤
Michel et al 2002a[126]	Medicine	Undergraduate	NR	28/56	PBL	LBL	①④
Michel et al 2002b[126]	Medicine	Undergraduate	NR	40/102	PBL	LBL	①④
Antepohl et al 1999[127]	Medicine	Undergraduate	NR	63/60	PBL	LBL	①③
Cheng et al 2021[128]	Medicine	Undergraduate	NR	124/120	PBL+CBL	LBL	①
Brinkman et al 2021[129]	Medicine	Undergraduate	NR	54/36	PBL	LBL	①④
MacDougall et al 2017[130]	Pharmacy	Undergraduate	/	360//360	AL	LBL	①
Tripathi et al 2015[131]	Medicine	Undergraduate	NSSD	45/45	AL	LBL	①⑤
Sumanasekera et al 2020[132]	Pharmacy	Undergraduate	NSSD	156/91	AL	LBL	①
Kennedy et al 2019[133]	Pharmacy	Undergraduate	NR	150/150	AL	LBL	①
Wei et al 2019[134]	Pharmacy	Undergraduate	NSSD	45/42	BOPPPS	LBL	④
Wang et al 2022[135]	Pharmacy	Junior college student	NSSD	30/30	BOPPPS	LBL	④
Zhang et al 2016[136]	Pharmacy	Undergraduate and Graduate	NR	60/60	EBM	LBL	①③⑤
Long et al 2011[137]	Pharmacy	Undergraduate	NSSD	80/80	EBM	LBL	①③
Song et al 2020[138]	Nursing	Junior college student	NSSD	120/120	MTM	LBL	①
Wang QY et al 2021[139]	Medicine	Undergraduate	NSSD	58/60	MTM	LBL	①
Zhao et al 2021[140]	Medicine	Junior college student	NSSD	102/105	MTM	LBL	①④
Yang XY(2) et al 2021[141]	Medicine	Undergraduate	NR	30/30	MTM	LBL	①⑤
Zhao J et al 2019[142]	Medicine	Junior college student	NSSD	78/85	MTM	LBL	①

Xing et al 2020[143]	Medicine	Junior college student	NSSD	55/55	MTM	LBL	①⑤
Yu et al 2020[144]	Medicine	Junior college student	NSSD	30/30	MTM	LBL	①②⑤
Sukhlecha et al 2016[145]	Pharmacy	Undergraduate	NSSD	65/65	AL	LBL	①
Liu JM et al 2015[146]	Medicine	Junior college student	NSSD	108/109	STM	LBL	①④
Jiang et al 2016[147]	Nursing	Junior college student	NR	20/15	STM	LBL	①
Gao et al 2021[148]	Medicine	Junior college student	NSSD	50/50	STM	LBL	①④
Wei et al 2009[149]	Pharmacy	Junior college student	NSSD	138/107	STM	LBL	①
He et al 2012[150]	Nursing	Junior college student	NSSD	50/48	TDL	LBL	①③

AL, active learning; BOPPPS, bridge-in, objective, pre-assessment, participatory learning, post-assessment, and summary; CBL, case-based learning; CoBL, computer-based learning; Cont, control; EBM, evidence-based medicine; FC, flipped classrooms; Interv, intervention; LBL, lecture based learning; MC, micro classrooms; MTM, mixed teaching mode; NR, not reported; NSSD, no statistically significant difference in baseline data; PBL, problem-based learning; STM, scaffolding teaching method; TBL, team-based learning; TDL, task driven learning. ① The theoretical test score. ② The experimental test score. ③ The subjective test score. ④ The satisfaction score. ⑤ The proportion of satisfaction.

[1] S. Sajjad and A. Gowani, “Introducing a flipped classroom in a pharmacology course,” *Br J Nurs*, vol. 30, no. 5, Art. no. 5, 2021, doi: 10.12968/bjon.2021.30.5.296.

[2] K. Lockman, S. T. Haines, and M. L. McPherson, “Improved Learning Outcomes After Flipping a Therapeutics Module: Results of a Controlled Trial,” *Acad Med*, vol. 92, no. 12, Art. no. 12, Dec. 2017, doi: 10.1097/ACM.0000000000001742.

[3] Y.-Y. Wu *et al.*, “Application and Evaluation of the Flipped Classroom Based on Micro-Video Class in Pharmacology Teaching.,” *Front Public Health*, vol. 10, p. 838900, 2022, doi: 10.3389/fpubh.2022.838900.

[4] M. M. El-Banna, M. Whitlow, and A. M. McNelis, “Flipping around the classroom: Accelerated Bachelor of Science in Nursing students’ satisfaction and achievement.,” *Nurse Educ Today*, vol. 56, pp. 41–46, Sep. 2017, doi: 10.1016/j.nedt.2017.06.003.

[5] A. Guo, “A Contrastive Study of Traditional Teaching and Flipped Classroom Teaching for Pharmacology in Secondary Vocational Schools,” *The Science Education Article Collects*, no. 10, Art. no. 10, 2018, doi: 10.16871/j.cnki.kjwhc.2018.10.052.

[6] C. Wang, Q. Wang, Z. Huang, L. Wang, and Y. Chen, “Application and evaluation of flipped classroom learning model in clinical pharmacology teaching for rural doctors under the new media environment,” *Journal of Community Medicine*, vol. 15, no. 13, Art. no. 13, 2017.

[7] T. Liang, L. Shi, D. Yuan, and S. O. Pharmacy, “Practice and Thinking About Moso Teach in Pharmacology Teaching,” *China Continuing Medical Education*, vol. 11, no. 22, pp. 31–33, 2019.

[8] Z. Wang, X. Guo, B. Shen, and J. Li, “Reconstruction of Content and Innovation of Pattern for Clinical Pharmacology Teaching under the Background of Precision Medicine,” *China Pharmacist*, vol. 23, no. 2. pp. 396–397, 2020.

[9] R. Ge, F. Yang, and Q. Zheng, “Application of Flipping Classroom Based on e-Teaching Platform in Experimental Teaching of Traditional Chinese Medicine Pharmacology,” *Education Teaching Forum*, no. 12, Art. no. 12, 2019.

[10] J. Wang, G. Huang, W. Li, and J. Pan, “Practice of Flipped Classroom Based on Blue Ink Cloud Class in Pharmacological Experiment Teaching,” *Guangdong Chemical Industry*, vol. 47, no. 01, Art. no. 01, 2020.

[11] H. Fu, L. Zhou, H. Xia, D. Qin, and D. Xu, "Effect of mixed pharmacology teaching mode based on 'flipped class- room'under the background of 'internet+,'" *China Modern Medicine*, vol. 29, no. 24. pp. 134–137, 2022.

[12] X. Zhao *et al.*, "Using Flipping Classroom & PBL in the teaching of Clinical Pharmacology," *China Higher Medical Education*, no. 5. pp. 113–114, 2019.

[13] T. H. Wong, E. J. Ip, I. Lopes, and V. Rajagopalan, "Pharmacy students' performance and perceptions in a flipped teaching pilot on cardiac arrhythmias," *Am J Pharm Educ*, vol. 78, no. 10, Art. no. 10, 2014, doi: 10.5688/ajpe7810185.

[14] B. Zhao, N. Zhao, X. Ma, and Z. Lu, "护理专业药理学实验翻转课堂模式教学设计与效果研究," *Journal of Henan Medical College*, vol. 30, no. 03, Art. no. 03, 2018.

[15] H. Fan, L. He, M. Shao, Y. Lai, Q. Lu, and Y. Wang, "基于翻转课堂的混合式教学模式在药理学教学中的应用," *China Journal of Multimedia & Network Teaching*, no. 03, Art. no. 03, 2020.

[16] H. Wang, Y. Ji, Z. Liang, and L. Chen, "基于微课和"翻转课堂"结合模式在药理学教学中的应用," *Self Care*, no. 12, pp. 245–246, 2021.

[17] T. Ding, H. Ma, and Q. Lv, "基于中国大学 mooc 平台的 spoc 翻转课堂在临床药理学教学中的应用," *Chemical Industry Times*, vol. 36, no. 8. pp. 45–47, 2022.

[18] T. Jia, "药理学教学中翻转课堂教学模式的应用探讨," *XINJIAOYUSHIDAIDIANZIZAZHI(JIAOSHIBAN)*, no. 47. p. 209, 2020.

[19] M. Bao, Y. Xia, C. Chen, H. Li, and X. Huang, "MBBS 留学生药理学翻转课堂的探索与实践," *Basic Medical Education*, vol. 20, no. 10, Art. no. 10, 2018, doi: 10.13754/j.issn2095-1450.2018.10.27.

[20] X. Yang, Z. Guo, J. Jiang, S. Sun, Z. Xie, and X. Lei, "翻转课堂联合超星学习通在医学检验专业药理学教学中的应用," *Journal of Higher Education*, no. 7, pp. 108–111, 2021.

[21] Z. Qin and X. Liu, "Exploration of Setting up a Comprehensive and Innovative Experimental Course of Pharmacology of Chinese Materia Medica," *Pharmaceutical Education*, vol. 36, no. 4, Art. no. 4, 2020.

[22] J. Ma, J. Wang, R. Lv, L. Gao, and M. Niu, "Application of Flipped Classroom Teaching Mode Combining Micro-Class and PBL in Pharmacology Teaching," *Journal of HuangHe S&T College*, vol. 24, no. 05, Art. no. 05, 2022, doi: 10.19576/j.issn.2096-790X.2022.05.021.

[23] W. Song, S. U. Xin, Z. Liu, L. Wei, and Y. Zhang, "Servation on the Application Effect of Micro Lesson Combined with PBL Teaching Method in Pharmacology Teaching," *Journal of Educational Institute of Jilin Province*, vol. 34, no. 6, pp. 184–186, 2018.

[24] X. Qin and T. Wang, "Application of PBL Teaching Method Combined with Micro-class in Pharmacology Teaching," *Medical Information*, vol. 33, no. 11. pp. 12–13, 2020.

[25] Y. Bai, M. Gao, Y. Wang, and H. Wang, "Application of micro-lectures in teaching clinical pharmacology," *Basic Medical Education*, vol. 20, no. 3, pp. 225–227, 2018.

[26] 杨秀兰, "浅析微课在高职护理专业药理学教学中的应用," *Health Vocational Education*, vol. 34, no. 1, Art. no. 1, 2016.

[27] L. Yang, S. Chen, and N. Zhang, "Using participatory teaching method in the experimental pharmacology," *China Higher Medical Education*, no. 10, Art. no. 10, 2021.

[28] 阮耀祥, “微课联合视频反馈教学法在药理实验教学中的应用——以常用实验动物技术为例,” *Health Vocational Education*, vol. 37, no. 12, Art. no. 12, 2019.

[29] J. Fan, M. Fan, Y. Zhao, X. Shi, and D. O. Pharmacy, “Construction of joint teaching mode based on WeChat public platform and divided classes and its application in Clinical Pharmacy teaching,” *China Medical Education Technology*, vol. 33, no. 5, pp. 579–582, 2019.

[30] L. Qin, Y. Mo, and Z. Shen, “微课嵌入式教学法在《药理学》教学中的应用——以毛果芸香碱药理作用机制为例,” *Comparative study of cultural innovation*, vol. 2, no. 5. pp. 183–184, 2018.

[31] G. Kaur *et al.*, “Case-Based Learning as an Effective Tool in Teaching Pharmacology to Undergraduate Medical Students in a Large Group Setting.,” *J Med Educ Curric Dev*, vol. 7, p. 2382120520920640, Dec. 2020, doi: 10.1177/2382120520920640.

[32] M. B. Vora and C. J. Shah, “Case-based learning in pharmacology: Moving from teaching to learning.,” *Int J Appl Basic Med Res*, vol. 5, no. Suppl 1, pp. S21-23, Aug. 2015, doi: 10.4103/2229-516X.162259.

[33] S. Li, B. Yu, and J. Yue, “Case-oriented self-learning and review in pharmacology teaching,” *Am J Med Sci*, vol. 348, no. 1, Art. no. 1, Jul. 2014, doi: 10.1097/MAJ.0000000000000197.

[34] S. K. Kamat, P. A. Marathe, T. C. Patel, Y. C. Shetty, and N. N. Rege, “Introduction of case based teaching to impart rational pharmacotherapy skills in undergraduate medical students,” *Indian J Pharmacol*, vol. 44, no. 5, Art. no. 5, 2012, doi: 10.4103/0253-7613.100400.

[35] U. K. Chiranjeevi, V. Gedela, and H. J. G. Rao, “A comparative study of case-based learning with conventional teaching in undergraduate training of pharmacology,” *Natl. J. Physiol. Pharm. Pharmacol.*, vol. 12, no. 5, Art. no. 5, 2022, doi: 10.5455/njppp.2022.12.02065202208032022.

[36] A. Xia *et al.*, “Practice and evaluation of pharmacology case-based learning in teaching antihypertensive drugs,” *Basic Medical Education*, vol. 19, no. 2, pp. 98–101, 2017.

[37] M. Chen, L. Ge, X. Li, M. Liu, and X. Huang, “Appliation of Case-discussion-method in Lessons of Pharmacology,” in *Proceedings of the 2015 International Forum on Higher Education*, 珠海, 2015, pp. 252–254. [Online]. Available: https://d.wanfangdata.com.cn/conference/ChZDb25mZXJlbmNlTmV3UzIwMjIxMTE3Egc5NDUyNjUzGgh4amUzM3Q5aQ%3D%3D

[38] J. H. Wang, S. M. Mao, and B. Kang, “Probe into case analysis model in teaching of clinical pharmacology,” *China Higher Medical Education*, no. 03, pp. 83–84, 2012.

[39] H. Yang, X. Du, H. Cong, Y. Wang, and Y. Lin, “Using research-oriented case study in pharmacology,” *China Higher Medical Education*, no. 9 %V. pp. 115–116, 2020.

[40] X. Li and L. Zheng, “案例分析教学法在临床专业药理学教学中的效果观察,” *BAOJIANWENHUI*, vol. 23, no. 23, Art. no. 23, 2022.

[41] Y. Chen, “案例教学法对提高临床药理学教学质量的影响,” *Journal of North Pharmacy*, vol. 11, no. 06, Art. no. 06, 2014.

[42] X. Liu, S. Chen, and G. Zhao, “案例教学法用于临床药理学教学的效果评价,” *China Pharmaceuticals*, no. 14, Art. no. 14 %V, 2015.

[43] Y. Yuan, R. Gu, and S. Ran, “案例教学法在儿科临床药理教学中的体会,” *Chongqing Medicine*, vol. 42, no. 21, Art. no. 21, 2013.

[44] L. Sui, Y. Gao, and B. Zhao, “案例教学法在护理专业药理课堂上的应用,” 护理实践与研究, vol. 6, no. 05, Art. no. 05, 2009.

[45] W. Huang, “案例教学法在临床医学专科药理学教学中的体会,” *scientific and technological innovationInformation*, no. 10, Art. no. 10, 2017.

[46] C. Zheng, “案例教学法在药理学抗高血压药教学中的应用,” *DA JIAN KANG*, no. 35, Art. no. 35 %V, 2020.

[47] L. Song, W. Zheng, and C. Guo, “案例式教学法在临床药理学各论理论教学中的实践,” *China Higher Medical Education*, no. 11, Art. no. 11, 2010.

[48] H. Wang, Y. Fu, Z. Han, and L. Yang, “案例式教学在药理学大班理论教学中的应用和评价,” *Journal of North Pharmacy*, no. 11 %V. pp. 169–169, 2014.

[49] M. Yin, “工学结合模式下药理学案例教学法探析,” *Modern Medicine & Health*, vol. 29, no. 8. pp. 1260–1261, 2013.

[50] Y. Zou, “论案例教学法在儿科临床药理教学中的体会,” *Innovation Education*, no. 11, Art. no. 11, 2014.

[51] Y. Wang, Y. Liu, M. Zhang, J. Li, and M. Chen, “药理学教学中 CBL 教学法的应用,” *JOURNAL OF SHANXI MEDICAL UNIVERSITY(PRECLINICAL MEDICAL EDUCATION EDITION)*, vol. 12, no. 08, Art. no. 08, 2010.

[52] H. Song, X. Xue, and D. Liu, “药学专业药理学教学中案例教学法的运用研究,” *Science & Technology Information*, no. 12. pp. 348–349, 2014.

[53] P. Liu, Y. Bai, and S. Pan, “中医院校药理课案例教学法初探,” in *Scientific Times - Proceedings of 2014 Scientific and Technological Innovation and Enterprise Management Symposium (Scientific and Technological Innovation)*, 2014, p. 86+88. Accessed: Nov. 18, 2022. [Online]. Available: https://kns.cnki.net/kcms/detail/detail.aspx?dbcode=CPFD&dbname=CPFD0914&filename=KJQY201404001083&uniplatform=NZKPT&v=8tUIa6WoIN-tymAu5DirGiV7FhB5L6c46MlG8st72UVmjZiI9LzfJ9X1b1aC0dYD8HAJM84dleE%3d

[54] G. Wang, Y. Zhang, W. Jiang, and D. O. Pharmacy, “Application of the CBL teaching mode in pharmacology teaching for higher vocational nursing specialty,” *China Medical Education Technology*, vol. 31, no. 3, pp. 350–353, 2017.

[55] C. Yang, X. Hu, Q. Gao, Y. Miao, H. Wang, and W. Gao, “CBL 与传统式教学法相结合在药理教学中的应用,” *The Journal of Medical Theory and Practice*, vol. 30, no. 08, Art. no. 08, 2017, doi: 10.19381/j.issn.1001-7585.2017.08.087.

[56] Z. G. Nie, C. Y. Gao, C. Y. Liang, S. Q. Wang, S. Wan, and Y. Chen, “Exploration and Thinking of Case Discussion in Pharmacology Teaching,” *Researches in Medical Education*, vol. 5, no. 10, pp. 923–924, 2006.

[57] R. D. Feldman, R. Schoenwald, and J. Kane, “Development of a computer-based instructional system in pharmacokinetics: efficacy in clinical pharmacology teaching for senior medical students.,” *J Clin Pharmacol*, vol. 29, no. 2, pp. 158–161, Feb. 1989, doi: 10.1002/j.1552-4604.1989.tb03306.x.

[58] J. C. MacFadyen, J. E. Brown, R. Schoenwald, and R. D. Feldman, “The effectiveness of teaching clinical pharmacokinetics by computer,” *Clin Pharmacol Ther*, vol. 53, no. 6, Art. no. 6, Jun. 1993, doi: 10.1038/clpt.1993.81.

[59] A. K. Hahne, R. Benndorf, P. Frey, and S. Herzig, “Attitude towards computer-based learning: determinants as revealed by a controlled interventional study.,” *Med Educ*, vol. 39, no. 9, pp. 935–943, Sep. 2005, doi: 10.1111/j.1365-2929.2005.02249.x.

[60] L. R. Joseph and S. R. Pillai, “Effectiveness of computer simulation versus chart-based learning in experimental pharmacology among undergraduate medical students,” *Natl. J. Physiol. Pharm. Pharmacol.*, vol. 11, no. 4, Art. no. 4, 2021, doi: 10.5455/njppp.2021.11.11324202001122020.

[61] H. X. Cui, “Exploration and practice of PBL bilingual teaching method in pharmacology,” *Journal of Qiqihar University of Medicine*, vol. 34, no. 22, pp. 3370–3371, 2013.

[62] W. Yu, C. Qiu, W. Song, Y. Zhang, and F. Cai, “案例药理学双语教学在普通本科院校中的探索与体会,” *Journal of Hubei University of Science and Technology*, vol. 36, no. 5. pp. 111–113, 2016.

[63] N. Huang *et al.*, “The Practice of English Case Teaching in Pharmacology Teaching,” *Journal of Kunming Medical University*, vol. 36, no. 5, pp. 171–173, 2015.

[64] X. Liu *et al.*, “Evaluations on the Teaching Effect by Application of LBL, PBL and CBL Teaching Model in Clinical Pharmacology Education,” *China Continuing Medical Education*, vol. 8, no. 18, pp. 8–10, 2016.

[65] S. D. Croteau, L. A. Howe, S. M. Timmons, L. Nilson, and V. G. Parker, “Evaluation of the effectiveness of ‘the village’: a pharmacology education teaching strategy,” *Nurs Educ Perspect*, vol. 32, no. 5, Art. no. 5, Oct. 2011, doi: 10.5480/1536-5026-32.5.338.

[66] H. Song, “CBL、PBL 在药理学教学改革中的应用体会,” *HEALTH MANAGEMENT*, no. 30, Art. no. 30 %V, 2021.

[67] S. Wang, “CBL、PBL 联合应用于药理学教学中的价值分析,” *Oriental Medicated Diet*, no. 16, Art. no. 16 %V, 2021.

[68] C. Li, J. Yang, H. Luo, and Y. Zeng, “The Practice and Exploration of Integrated Teaching Model of LBL, CBL and PBL in Pharmacology Teaching,” *The Science Education Article Collects*, no. 34, pp. 89–91, 2019.

[69] H. Li, Z. Sun, and W. Jin, “CBL 与 TBL 教学法在药理学教学中联合应用对高职医学生人文素质和学习态度的影响,” *Journal of Seeking Knowledge Guide*, no. 6. pp. 22–23, 2019.

[70] H. Li and Z. Sun, “CBL 与 TBL 教学法联合应用——对高职临床医学专业药理学课程教学的效果评价,” *Course Education Research*, no. 03, Art. no. 03, 2020.

[71] J. Ma, H. Y. Luo, Y. Yang, C. Li, Y. Yun, and J. Y. Yang, “Application and Evaluation of Combination of PBL/CBL/TBL Teaching Method in Anesthetic Pharmacology Experiment,” *Journal of Kunming Medical University*, 2017.

[72] N. Dai *et al.*, “Application of the Integrated Teaching Method of PBL and TBL Based on Cases in Pharmacology Teaching,” *Journal of Kunming Medical University*, vol. 37, no. 10, pp. 137–139.

[73] H. James, Y. I. Y. Tayem, K. a. J. Al Khaja, S. Veeramuthu, and R. P. Sequeira, “Prescription Writing in Small Groups as a Clinical Pharmacology Educational Intervention: Perceptions of Preclerkship Medical Students,” *J Clin Pharmacol*, vol. 56, no. 8, Art. no. 8, Aug. 2016, doi: 10.1002/jcph.692.

[74] S. Zhou and W. Dong, “TBL 结合虚拟实验教学在药理实验教学中的应用,” *Contemporary Education Research and Teaching Practice %@*, no. 16 %V, Art. no. 16 %V, 2020.

[75] H. Xia, W. Ding, L. Zhang, and M. Yang, “TBL 教学模式在药理学实验中的应用效果,” *Journal of Heze Medical College*, vol. 34, no. 3. pp. 82–84, 2022.

[76] X. Wu, H. Zhang, and Q. Xue, “Practice of TBL teaching in pharmacology teaching for foreign students,” *Chinese Journal of Medical Education Research*, vol. 16, no. 02, pp. 152–155, 2017.

[77] X. Li, B. Zeng, F. Deng, L. Xiao, and F. Chen, “解析 TBL 教学法在药理学教学中的应用,” *Education for Chinese After-school*, no. z1. pp. 229–229, 2013.

[78] G. A. Carrasco, K. C. Behling, M. Gentile, B. D. Fischer, and T. N. Ferraro, “Effectiveness of a Team-Based Learning exercise in the learning outcomes of a medical pharmacology course: insight from struggling students,” *Naunyn-Schmiedeberg’s Arch. Pharmacol.*, vol. 394, no. 9, Art. no. 9, 2021, doi: 10.1007/s00210-021-02093-3.

[79] D. S. Palappallil, H. K. N. Sankar, A. Retnayyan, and S. Radhakrishnan, “Effectiveness of case-based learning, task-based learning, and didactic lectures on teaching personal drug concept among medical undergraduates,” *Natl. J. Physiol. Pharm. Pharmacol.*, vol. 9, no. 5, Art. no. 5, 2019, doi: 10.5455/njppp.2019.9.0308619032019.

[80] J. McCartney and S.-A. Boschmans, “Evaluation of an intervention to support the development of clinical problem solving skills during a hospital-based experiential learning program for South African pharmacy students,” *Currents Pharm. Teach. Learn.*, vol. 12, no. 5, Art. no. 5, 2020, doi: 10.1016/j.cptl.2020.01.016.

[81] M. M. El-Banna, M. Whitlow, and A. M. McNelis, “Improving Pharmacology Standardized Test and Final Examination Scores Through Team-Based Learning,” *Nurse Educ*, vol. 45, no. 1, Art. no. 1, Feb. 2020, doi: 10.1097/NNE.0000000000000671.

[82] N. K. Zgheib, J. A. Simaan, and R. Sabra, “Using team-based learning to teach pharmacology to second year medical students improves student performance.,” *Med Teach*, vol. 32, no. 2, Art. no. 2, 2010, doi: 10.3109/01421590903548521.

[83] A. M. Persky, “The impact of team-based learning on a foundational pharmacokinetics course,” *Am J Pharm Educ*, vol. 76, no. 2, Art. no. 2, Mar. 2012, doi: 10.5688/ajpe76231.

[84] D.-H. Kim, J.-H. Lee, and S. A. Kim, “The pharmacology course for preclinical students using team-based learning.,” *Korean J Med Educ*, vol. 32, no. 1, pp. 35–46, Mar. 2020, doi: 10.3946/kjme.2020.151.

[85] T. Nguyen, E. Wong, and A. Pham, “Incorporating Team-Based Learning Into a Physician Assistant Clinical Pharmacology Course.,” *J Physician Assist Educ*, vol. 27, no. 1, Art. no. 1, Mar. 2016, doi: 10.1097/jpa.0000000000000061.

[86] W. Dong, “基于微课的 TBL 教学法在本科药理学实验教学中的应用,” *Health Vocational Education*, vol. 37, no. 17, Art. no. 17, 2019.

[87] S. Xiang and Y. Yang, “高等职业院校护理专业药理学教学应用 PBL 教学法的效果分析,” *JIN RI HU BEI*, no. 6, pp. 125–126, 2015.

[88] L. Liu, “高职护理专业药理学课程 PBL 教学和传统教学的比较研究,” *Journal of North Pharmacy*, no. 2, pp. 137–137, 2014.

[89] X. Wang and S. Wang, “高职护理专业药理学 PBL 教学法的效果评价与分析,” *Education and Vocation*, no. 18, Art. no. 18, 2012, doi: 10.13615/j.cnki.1004-3985.2012.18.002.

[90] C. Yang and W. Jin, “The application of problem-based learning in the teaching of nursing pharmacology,” *CHINA MEDICAL HERALD*, vol. 5, no. 28. pp. 82–83, 2008.

[91] T. Zhao, “护理药理教学中应用 PBL 教学法初探,” *Healthmust-Readmagazine*, no. 25, pp. 253–254, 2021.

[92] F. Yang, “探究 PBL+LBL 双轨教学模式在药理教学中的应用对策,” *Diet Health*, no. 7, pp. 160–161, 2015.

[93] S. Li, F. Tian, L. Shao, D. Zhai, and S. Liu, “Application of Virtual Simulation Experiment Combined with PBL Teaching in Pharmacology Experiments,” *China Educational Technology & Equipment*, no. 12, Art. no. 12, 2020.

[94] T. Jia, “药理学教学中 PBL 教学法的应用效果研究,” *New Education Era*, no. 20, p. 191, 2021.

[95] J. Li, J. Chen, J. Miao, and Y. Feng, “The pharmacological experiment integration teaching method,means of reform and Practice,” *China Pharmaceuticals*, no. 16, pp. 11–13, 2014.

[96] T. Liang, L. Zhou, D. Yuan, L. Shi, and D. O. Pharmacology, “Study on the Teaching Effect of PBL in the Teaching of Pharmacology,” *Chinese Medicine Modern Distance Education of China*, vol. 15, no. 13, pp. 14–15, 2017, doi: 10.3969/ j.issn.1672-2779.2017.13.007.

[97] W. Zhao, X. Lan, K. Zhu, and Y. Gong, “Effect analysis of pharmacology PBL teaching,” *China Medical Herald*, vol. 11, no. 7, pp. 135–137, 141, 2014.

[98] S. Xiang and Y. Yang, “以问题为基础的互动式教学在药理学教学中的应用效果评价,” *JIN RI HU BEI*, no. 6, pp. 99–100, 2015.

[99] S. Wang, Y. Wu, J. Wang, and X. Qian, “Investigation and study of pharmacological teaching method reform in undergraduate,” *CHINESE JOURNAL OF MEDICAL EDUCATION*, vol. 28, no. 2. pp. 58–59, 2008.

[100] B. Cao, S. Li, and H. He, “The application of problem-based learning in the clinical tumor pharmacology teaching,” *Chinese Journal of Medical Education*, vol. 31, no. 3. pp. 405-406,428, 2011.

[101] R. Zhao, Z. Yang, D. Yan, P. Sun, and T. Zhang, “‘以问题为中心的学习’在中药药理学教学过程中的应用效果分析,” *China Higher Medical Education*, no. 04, pp. 51–52, 2015.

[102] W. Chen and Q. Peng, “‘以问题为中心教学法’在药理学教学中的研究与实践,” *JOURNAL OF YICHUN UNIVERSITY*, vol. 29, no. 4. p. 137,169, 2007.

[103] S. L. Liang, L. I. Qing-Yao, S. L. Gan, X. G. Zhou, W. Y. Liu, and F. G. Yan, “Problem- centered teaching approach in pharmacology teaching,” *Journal of Jinggangshan University*, no. 04, pp. 121–122, 127, 2008.

[104] Y. Wang *et al.*, “以 PBL 为导向的分阶段教学模式在药理实验教学中的应用,” *Science & Technology Vision*, no. 12, Art. no. 12, 2017, doi: 10.19694/j.cnki.issn2095-2457.2017.12.021.

[105] W. Chen, “The Exploration and Application of Problem-based Learning in the Teaching of Pharmacology and Application of Chinese Materia Medica,” *Chinese Medicine Modern Distance Education of China*, vol. 15, no. 17, pp. 34–35, 60, 2017.

[106] S. K. Miller, “A comparison of student outcomes following problem-based learning instruction versus traditional lecture learning in a graduate pharmacology course.,” *J Am Acad Nurse Pract*, vol. 15, no. 12, pp. 550–556, Dec. 2003, doi: 10.1111/j.1745-7599.2003.tb00347.x.

[107] P. Sengupta and T. Sur, “Effectiveness of Whatsapp as a teaching learning tool for problem based learning in pharmacology: A Quasi-experimental study,” *J. Clin. Diagn. Res.*, vol. 15, no. 11, Art. no. 11, 2021, doi: 10.7860/JCDR/2021/51256.15568.

[108] T. Liang, L. Zhou, D. Yuan, L. Shi, and D. O. Pharmacology, “Study on the Teaching Effect of LBL Combined With PBL in Pharmacology Teaching,” *China Continuing Medical Education*, vol. 9, no. 11, pp. 40–42, 2017, doi: 10.3969/j.issn.1674-9308.2017.11.018.

[109] S. Herzig, R.-M. Linke, B. Marxen, U. Börner, and W. Antepohl, “Long-term follow up of factual knowledge after a single, randomised problem-based learning course.,” *BMC Med Educ*, vol. 3, p. 3, Apr. 2003, doi: 10.1186/1472-6920-3-3.

[110] L. Chen *et al.*, “PBL＋LBL 教学法在留学生药理学教学中的应用,” *Basic Medical Education*, no. 9 %V, pp. 796-797,798, 2015.

[111] Y. Fu, L. Yang, and X. Li, “Teaching Model of PBL Combined with LBL Used in Pharmacology for Seven Years Students of Traditional Chinese Medicine,” *Journal of Liaoning University of Traditional Chinese Medicine*, vol. 18, no. 8, pp. 118–120, 2016.

[112] Y. Yang and S. Luo, “PBL 教学法在高职高专药理学教学中的应用分析,” *Kaoshi Zhoukan*, no. 54, Art. no. 54, 2018.

[113] S. Guo, Y. Wang, and Z. Li, “Application of PBL Teaching Method In ‘pharmacology’ Teaching for Vocational college nursing students,” *Chinese Nursing Research*, vol. 27, no. 26. pp. 2935–2936, 2013.

[114] W. Li and H. Chi, “PBL 教学法在高职药理学教学中应用与评价,” *Science & Technology Vision*, no. 15, Art. no. 15, 2014, doi: 10.19694/j.cnki.issn2095-2457.2014.15.103.

[115] L. Zhou, S. Luo, D. Qin, and L. Cui, “PBL 教学法在临床药理学病案讨论课中的应用研究,” *China Higher Medical Education*, no. 11. pp. 13–14, 2012.

[116] W. Huang, “PBL 教学法在临床医学专科药理学教学中的应用与评价,” *Scientific and Technological Innovation*, no. 09, Art. no. 09, 2017.

[117] L. Li, J. Chang, Y. Zhou, and Z. Gao, “PBL 教学法在农业院校中药学专业《中药药理学》实验教学中的应用,” *China Medicine and Pharmacy*, no. 24 vo 1, pp. 137–138, 2011.

[118] H. P. Song, H. Y. Huang, X. Cai, R. Zeng, and Z. Q. Wang, “Practice of PBL Teaching Mode in the Teaching of Pharmacology,” *Journal of Higher Education Research*, vol. 38, no. 03, pp. 113–117, 2015.

[119] B. Qin, T. Yu, H. Zhang, and X. Liang, “Attempt of problem-based learning in anesthetic and pharmacology,” *CHINA MEDICAL HERALD*, vol. 9, no. 23. pp. 140–141, 2012.

[120] Y. Jia, “PBL 教学在专科学校的药理学教学中的应用,” *Guide of China Medicine*, vol. 11, no. 25, Art. no. 25, 2013, doi: 10.15912/j.cnki.gocm.2013.25.008.

[121] J. Gao, “PBL 结合病案讨论式教学法在药理学教学中的应用,” *Health Vocational Education*, vol. 33, no. 1, Art. no. 1, 2015.

[122] M. Yang, X. Sun, Y. Xu, and H. Luan, “PBL 结合 LBL 教学法在麻醉专业药理学教学中的改革探索,” *China Higher Medical Education*, no. 5. pp. 119–120, 2018.

[123] Z. Song *et al.*, “PBL 与 LBL 相结合的教学方法在药理学整合教学中的效果评价,” *China Higher Medical Education*, no. 04, Art. no. 04, 2008.

[124] G. Li and B. Zhang, “PBL 在我校中药药理学本科教学中的应用,” *Journal Of Inner Mongolia Medical University*, no. S1 vo 39, Art. no. S1 vo 39, 2017, doi: 10.16343/j.cnki.issn.2095-512x.2017.s1.033.

[125] Y. Huo, J. Yang, and Z. Gong, “PBL 在药理实验教学中的探索与实践,” *The Journal of Medical Theory and Practice*, vol. 29, no. 14, Art. no. 14, 2016, doi: 10.19381/j.issn.1001-7585.2016.14.093.

[126] M. C. Michel, A. Bischoff, M. Zu Heringdorf, D. Neumann, and K. H. Jakobs, “Problem- vs. lecture-based pharmacology teaching in a German medical school,” *Naunyn Schmiedebergs Arch Pharmacol*, vol. 366, no. 1, Art. no. 1, Jul. 2002, doi: 10.1007/s00210-002-0570-x.

[127] W. Antepohl and S. Herzig, “Problem-based learning versus lecture-based learning in a course of basic pharmacology: a controlled, randomized study.,” *Med Educ*, vol. 33, no. 2, Art. no. 2, Feb. 1999, doi: 10.1046/j.1365-2923.1999.00289.x.

[128] L.-F. Cheng, J.-S. Kang, and Q. Xu, “Research on the constructing the holistic thinking mode and teaching effect of pharmacology,” *Indian J. Pharm. Educ. Res.*, vol. 55, no. 4, Art. no. 4, 2021, doi: 10.5530/ijper.55.4.194.

[129] D. J. Brinkman, T. Monteiro, E. C. Monteiro, M. C. Richir, M. A. van Agtmael, and J. Tichelaar, “Switching from a traditional undergraduate programme in (clinical) pharmacology and therapeutics to a problem-based learning programme,” *Eur. J. Clin. Pharmacol.*, vol. 77, no. 3, Art. no. 3, 2021, doi: 10.1007/s00228-020-03027-3.

[130] C. MacDougall, “A Novel Teaching Tool Combined With Active-Learning to Teach Antimicrobial Spectrum Activity.,” *Am J Pharm Educ*, vol. 81, no. 2, p. 25, Mar. 2017, doi: 10.5688/ajpe81225.

[131] R. K. Tripathi, P. V. Sarkate, S. V. Jalgaonkar, and N. N. Rege, “Development of active learning modules in pharmacology for small group teaching.,” *Educ Health (Abingdon)*, vol. 28, no. 1, pp. 46–51, Apr. 2015, doi: 10.4103/1357-6283.161851.

[132] W. Sumanasekera, C. Turner, K. Ly, P. Hoang, T. Jent, and T. Sumanasekera, “Evaluation of multiple active learning strategies in a pharmacology course,” *Currents Pharm. Teach. Learn.*, vol. 12, no. 1, pp. 88–94, 2020, doi: 10.1016/j.cptl.2019.10.016.

[133] D. R. Kennedy, “Redesigning a Pharmacology Course to Promote Active Learning,” *Am J Pharm Educ*, vol. 83, no. 5, Art. no. 5, Jun. 2019, doi: 10.5688/ajpe6782.

[134] L. Wei, W. Song, S. Jiang, Y. Zhang, Z. Liu, and E. Zhu, “BOPPPS 教学模式在药理教学中的应用效果观察,” *China Journal of Multimedia & Network Teaching*, no. 05, Art. no. 05, 2019.

[135] W. Wang, L. Yuan, and Y. Liu, “BOPPPS 教学模式在药理教学中的应用效果评价,” *ORIENTAL MEDICATED DIET*, no. 6, Art. no. 6 %V, 2022.

[136] Y. Zhang *et al.*, “Feasibility analysis and effect evaluation of teaching model of pharmacological evidence based medicine in medical university,” *China Medicine and Pharmacy*, vol. 6, no. 10, pp. 22–25, 2016.

[137] M. Long, D. Yao, C. Li, and X. Bai, “循证医学理念与抛锚式教学法在药理学临床实践课程教学中的应用,” *PHARMACY TODAY*, vol. 21, no. 9, Art. no. 9, 2011.

[138] Q. Song, “Application of mixed teaching method in Nursing Pharmacology,” *Chinese & Foreign Entrepreneurs*, no. 20, Art. no. 20, 2020.

[139] Q. Wang, H. Sun, and J. Yang, "'点、线、面'教学法在药理学线上+线下混合教学中的应用," *China Higher Medical Education*, no. 06, Art. no. 06, 2021.

[140] B. Zhao, K. Wang, and X. Ma, "智慧学习环境下高职高专混合式教学模式在药理学课程中的研究与实践," *Health Vocational Education*, no. 01 vo 39, Art. no. 01 vo 39, 2021.

[141] X. Yang *et al.*, "基于 MOOC 联合雨课堂的药理学混合式教学模式探索与实践," *Modern Vocational Education*, no. 28, Art. no. 28, 2021.

[142] J. Zhao, "基于蓝墨云班课的高职药理学教学效果分析," *Industrial & Science Tribune*, vol. 18, no. 16. pp. 184–185, 2019.

[143] Z. Xing, "The Application of Super Star Learning Platform in Pharmacology Teaching —— Taking Antithyroid Drugs as an Example," *China Computer & Communication*, no. 19 vo 32, Art. no. 19 vo 32, 2020.

[144] W. Yu and J. Song, "基于课堂派的混合式学习模式在高职高专《药理学》教学中的应用研究," *Chinese Information*, no. 3 %V, Art. no. 3 %V, 2020.

[145] A. Sukhlecha, S. P. Jadav, T. R. Gosai, and D. Balusamy, "Student-led objective tutorials in Pharmacology: An interventional study.," *Indian J Pharmacol*, vol. 48, no. Suppl 1, Art. no. Suppl 1, Oct. 2016, doi: 10.4103/0253-7613.193310.

[146] J. Liu, F. Wang, M. Qiu, D. Jiang, and S. Xu, "支架式教学法在大专临床专业药理学教学中的应用," *Health Vocational Education*, vol. 33, no. 14, Art. no. 14 vo 33, 2015.

[147] G. Jiang, Y. Yang, and F. Wang, "支架式教学法在高职高专《护理药理学》的研究与应用," *Journal of Shandong Medical College*, vol. 38, no. 05, Art. no. 05 vo 38, 2016.

[148] F. Gao, "Application of Scaffolding Teaching Method in Clinical Pharmacology Experiment Teaching," *Smart Healthcare*, vol. 7, no. 12. pp. 156–158, 2021.

[149] C. Wei, J. Wu, and J. Liu, "支架式教学法在药理学课程中的应用," *Chinese Vocational and Technical Education*, no. 32, Art. no. 32, 2009.

[150] Y. He and X. Zhang, "Application of task-driven methods in pharmacology classroom teaching," *CHINA MEDICAL HERALD*, vol. 9, no. 12. pp. 162–163, 2012.

Table S3. The first ranking method in different specialties subgroups and their surface under the cumulative ranking (SUCRA) probabilities.

**Specialties**	**Medicine**	**Pharmacy**	**Nursing**
**The theoretical test score**			
Methods	PBL&CBL	FC	PBL
SUCRA probabilities	87.50%	91.45%	73.98%
**The satisfaction score**			
Methods	TBL	FC&BOPPPS	FC
SUCRA probabilities	82.02%	99.61%	74.33%
**The proportion of satisfaction**			
Methods	FC	TBL	FC
SUCRA probabilities	75.62%	99.72%	74.92%

BOPPPS, bridge-in, objective, pre-assessment, participatory learning, post-assessment, and summary; CBL, case-based learning; FC, flipped classrooms; PBL, problem-based learning; TBL, team-based learning.

2 Supplementary Figures

Study	Random sequence generation (selection bias)	Allocation concealment (selection bias)	Blinding of participants and personnel (performance bias)	Blinding of outcome assessment (detection bias)	Incomplete outcome data (attrition bias)	Selective reporting (reporting bias)	Other bias
Bao et al 2018	unclear	unclear	NA	unclear	low	low	low
Guo et al 2018	low	unclear	NA	unclear	low	low	low
Jia et al 2020	low	unclear	NA	unclear	low	low	low
Wang et al 2017	low	unclear	NA	unclear	low	low	low
Liang et al 2019	low	unclear	NA	unclear	low	low	low
Wang ZC et al 2020	unclear	unclear	NA	unclear	low	low	low
Ge et al 2019	unclear	unclear	NA	unclear	low	low	low
Ding et al 2022	low	unclear	NA	low	low	low	low
Wang H et al 2021	unclear	unclear	NA	unclear	low	low	low
Wang JN et al 2020	low	unclear	NA	unclear	low	low	low
Fan et al 2020	unclear	unclear	NA	unclear	low	low	low
Zhao et al 2018	unclear	unclear	NA	low	low	low	low
Fu et al 2022	unclear	unclear	NA	unclear	low	low	low
Zhao XM et al 2019	unclear	unclear	NA	unclear	low	low	low
Yang XY et al 2021	low	unclear	NA	unclear	low	low	low
Wong et al 2014	unclear	unclear	NA	low	low	low	low
Sajjad et al 2021	unclear	unclear	NA	unclear	low	low	low
Lockman et al 2017	unclear	unclear	NA	low	low	low	low
Wu et al 2022	low	unclear	NA	unclear	low	low	low
El-Banna et al 2017	unclear	unclear	NA	unclear	low	low	low
Qin Z et al 2020	low	unclear	NA	unclear	low	low	low
Ma et al 2022	low	unclear	NA	unclear	low	low	low
Song et al 2018	unclear	unclear	NA	unclear	low	low	low
Qin X et al 2020	low	unclear	NA	unclear	low	low	low
Bai et al 2018	low	unclear	NA	low	low	low	low
Yang et al 2016	unclear	unclear	NA	unclear	low	low	low
Yang LJ et al 2021	low	unclear	NA	unclear	low	low	low
Ruan et al 2019	unclear	unclear	NA	unclear	low	low	low
Fan et al 2019	low	unclear	NA	unclear	low	low	low
Qin et al 2018	low	unclear	NA	low	low	low	low
Li et al 2022	unclear	unclear	NA	unclear	low	low	low

Study	Random sequence generation (selection bias)	Allocation concealment (selection bias)	Blinding of participants and personnel (performance bias)	Blinding of outcome assessment (detection bias)	Incomplete outcome data (attrition bias)	Selective reporting (reporting bias)	Other bias
Chen et al 2014	unclear	unclear	NA	unclear	low	low	low
Liu XJ et al 2015	unclear	unclear	NA	unclear	low	low	low
Yuan et al 2013	low	unclear	NA	unclear	low	low	low
Sui et al 2009	unclear	unclear	NA	unclear	low	low	low
Huang W et al 2017	low	unclear	NA	low	low	low	low
Xia et al 2017	unclear	unclear	NA	unclear	low	low	low
Zheng et al 2020	unclear	unclear	NA	unclear	low	low	low
Song et al 2010	low	unclear	NA	unclear	low	low	low
Wang et al 2014	unclear	unclear	NA	low	low	low	low
Chen MJ et al 2015	unclear	unclear	NA	unclear	low	low	low
Yin et al 2013	low	unclear	NA	low	low	low	low
Wang JH et al 2012	low	unclear	NA	unclear	low	low	low
Zou et al 2014	low	unclear	NA	unclear	low	low	low
Wang et al 2010	unclear	unclear	NA	unclear	low	low	low
Song et al 2014	low	unclear	NA	unclear	low	low	low
Yang et al 2020	unclear	unclear	NA	unclear	low	low	low
Liu P et al 2014	unclear	unclear	NA	low	low	low	low
Wang GP et al 2017	unclear	unclear	NA	unclear	low	low	low
Yang et al 2017	low	unclear	NA	low	low	low	low
Nie et al 2006	unclear	unclear	NA	unclear	low	low	low
Kaur et al 2020	low	unclear	NA	unclear	low	low	low
Vora et al 2015	low	unclear	NA	low	low	low	low
Li S et al 2014	low	unclear	NA	low	low	low	low
Kamat et al 2012	low	unclear	NA	low	low	low	low
Chiranjeevi et al 2022	low	unclear	NA	low	low	low	low
Joseph et al 2021	low	unclear	NA	unclear	low	low	low
Cui et al 2013	low	unclear	NA	unclear	low	low	low
Yu et al 2016	low	unclear	NA	low	low	low	low
Huang et al 2015	unclear	unclear	NA	low	low	low	low
Liu et al 2016	unclear	unclear	NA	unclear	low	low	low
Croteau et al 2011	unclear	unclear	NA	low	low	low	low

Study	Random sequence generation (selection bias)	Allocation concealment (selection bias)	Blinding of participants and personnel (performance bias)	Blinding of outcome assessment (detection bias)	Incomplete outcome data (attrition bias)	Selective reporting (reporting bias)	Other bias
Song et al 2021	low	unclear	NA	unclear	low	low	low
Wang SC et al 2021	unclear	unclear	NA	unclear	low	low	low
Li C et al 2019	low	unclear	NA	unclear	low	low	low
Li HY et al 2019	low	unclear	NA	unclear	low	low	low
Li et al 2020	unclear	unclear	NA	low	low	low	low
Ma et al 2017	low	unclear	NA	low	low	low	low
Dai et al 2016	unclear	unclear	NA	low	low	low	low
James et al 2016	unclear	unclear	NA	unclear	low	low	low
Zhou et al 2020	low	unclear	NA	unclear	low	low	low
Xia et al 2022	unclear	unclear	NA	unclear	low	low	low
Wu et al 2017	unclear	unclear	NA	unclear	low	low	low
Li et al 2013	low	unclear	NA	unclear	low	low	low
Carrasco et al 2021	unclear	unclear	NA	unclear	low	low	low
Palappallil et al 2019	unclear	unclear	NA	low	low	low	low
McCartney et al 2020	unclear	unclear	NA	low	low	low	low
El-Banna et al 2020	low	unclear	NA	low	low	low	low
Zgheib et al 2010	unclear	unclear	NA	low	low	low	low
Persky et al 2012	low	unclear	NA	low	low	low	low
Kim et al 2020	unclear	unclear	NA	low	low	low	low
Nguyen et al 2016	unclear	unclear	NA	low	low	low	low
Dong et al 2019	unclear	unclear	NA	unclear	low	low	low
Xiang S et al 2015	low	unclear	NA	unclear	low	low	low
Liu LJ et al 2014	low	unclear	NA	low	low	low	low
Wang XL et al 2012	low	unclear	NA	unclear	low	low	low
Yang et al 2021	low	unclear	NA	unclear	low	low	low
Yang et al 2008	low	unclear	NA	low	low	low	low
Yang et al 2015	low	unclear	NA	low	low	low	low
Li et al 2020	low	unclear	NA	unclear	low	low	low
Jia et al 2021	low	unclear	NA	unclear	low	low	low
Li J et al 2014	unclear	unclear	NA	unclear	low	low	low
Liang T et al 2017	unclear	unclear	NA	low	low	low	low

Study	Random sequence generation (selection bias)	Allocation concealment (selection bias)	Blinding of participants and personnel (performance bias)	Blinding of outcome assessment (detection bias)	Incomplete outcome data (attrition bias)	Selective reporting (reporting bias)	Other bias
Zhao et al 2014	low	unclear	NA	unclear	low	low	low
Xiang S(2) et al 2015	unclear	unclear	NA	unclear	low	low	low
Wang et al 2008	unclear	unclear	NA	unclear	low	low	low
Cao et al 2011	low	unclear	NA	unclear	low	low	low
Zhao et al 2015	low	unclear	NA	low	low	low	low
Chen et al 2007	low	unclear	NA	low	low	low	low
Liang et al 2008	low	unclear	NA	low	low	low	low
Wang YL et al 2017	unclear	unclear	NA	unclear	low	low	low
Chen et al 2017	low	unclear	NA	low	low	low	low
Miller et al 2003	unclear	low	NA	low	low	low	low
Sengupta et al 2021	low	unclear	NA	unclear	low	low	low
Liang T(2) et al 2017	unclear	unclear	NA	unclear	low	low	low
Herzig et al 2003	low	unclear	NA	low	low	low	low
Chen L et al 2015	unclear	unclear	NA	unclear	low	low	low
Fu et al 2016	unclear	unclear	NA	unclear	low	low	low
Yang Y et al 2018	low	unclear	NA	low	low	low	low
Guo et al 2013	unclear	unclear	NA	low	low	low	low
Li W et al 2014	low	unclear	NA	low	low	low	low
Zhou et al 2012	low	unclear	NA	unclear	low	low	low
Huang W(2) et al 2017	low	unclear	NA	low	low	low	low
Li et al 2011	unclear	unclear	NA	unclear	low	low	low
Song et al 2015	unclear	unclear	NA	low	low	low	low
Qin et al 2012	low	unclear	NA	unclear	low	low	low
Jia et al 2013	unclear	unclear	NA	unclear	low	low	low
Gao et al 2015	unclear	unclear	NA	unclear	low	low	low
Yang M et al 2018	low	unclear	NA	unclear	low	low	low
Song et al 2008	low	unclear	NA	unclear	low	low	low
Li et al 2017	low	unclear	NA	low	low	low	low
Huo et al 2016	low	unclear	NA	low	low	low	low
Michel et al 2002	low	unclear	NA	unclear	low	low	low
Antepohl et al 1999	low	low	NA	low	low	low	low

Study	Random sequence generation (selection bias)	Allocation concealment (selection bias)	Blinding of participants and personnel (performance bias)	Blinding of outcome assessment (detection bias)	Incomplete outcome data (attrition bias)	Selective reporting (reporting bias)	Other bias
Cheng et al 2021	unclear	unclear	NA	unclear	low	low	low
Brinkman et al 2021	unclear	unclear	NA	unclear	low	low	low
MacDougall et al 2017	low	unclear	NA	low	low	low	low
Tripathi et al 2015	low	unclear	NA	low	low	low	low
Sumanasekera et al 202	unclear	unclear	NA	low	low	low	low
Kennedy et al 2019	unclear	unclear	NA	unclear	low	low	low
Feldman et al 1989	low	unclear	NA	unclear	low	low	low
MacFadyen et al 1993	low	unclear	NA	low	low	low	low
Hahne et al 2005	low	unclear	NA	low	low	low	low
Wei et al 2019	low	unclear	NA	unclear	low	low	low
Wang et al 2022	low	unclear	NA	unclear	low	low	low
Zhang et al 2016	low	unclear	NA	unclear	low	low	low
Long et al 2011	low	unclear	NA	low	low	low	low
Song et al 2020	unclear	unclear	NA	low	low	low	low
Wang QY et al 2021	low	unclear	NA	unclear	low	low	low
Zhao et al 2021	unclear	unclear	NA	unclear	low	low	low
Yang XY(2) et al 2021	low	unclear	NA	low	low	low	low
Zhao J et al 2019	unclear	unclear	NA	unclear	low	low	low
Xing et al 2020	low	unclear	NA	low	low	low	low
Yu et al 2020	low	unclear	NA	low	low	low	low
Sukhlecha et al 2016	low	unclear	NA	low	low	low	low
Liu JM et al 2015	unclear	unclear	NA	unclear	low	low	low
Jiang et al 2016	low	unclear	NA	low	low	low	low
Gao et al 2021	low	unclear	NA	unclear	low	low	low
Wei et al 2009	low	unclear	NA	low	low	low	low
He et al 2012	unclear	unclear	NA	unclear	low	low	low

NA: not applicable

Supplementary Figure 1. The individual assessment of risk of bias.
